# Supplementary material for: Oxidative photocatalysis on membranes triggers non-canonical pyroptosis
Source: Nat Commun. 2024 May 13;15:4025. doi: 10.1038/s41467-024-47634-5 (PMC11091103; doi:10.1038/s41467-024-47634-5)
Supplement: Supplementary file 1 — Supplementary Information [file 41467_2024_47634_MOESM1_ESM.docx]

**­**

Supplementary Information

**Oxidative photocatalysis on membranes triggers non-canonical pyroptosis**

Chaiheon Lee, Mingyu Park, W.C. Bhashini Wijesinghe, Seungjin Na, Chae Gyu Lee, Eunhye Hwang, Gwangsu Yoon, Jeong Kyeong Lee, Deok-Ho Roh, Yoon Hee Kwon, Jihyeon Yang, Sebastian A. Hughes, James E. Vince, Jeong Kon Seo^*^, Duyoung Min^*^, and Tae-Hyuk Kwon^*^

*To whom correspondence should be addressed:

jkseo6998@unist.ac.kr (J. K. Seo), [dymin@unist.ac.kr](mailto:dymin@unist.ac.kr) (D. Min), [kwon90@unist.ac.kr](mailto:kwon90@unist.ac.kr) (T-H. Kwon),

Contents

**Supplementary Method 1. Synthesis**

**Supplementary Method 2. Photophysical and electrochemical properties**

**Supplementary Method 3. Reactive oxygen species (ROS) generation**

**Supplementary Method 4. Photocatalytic oxidation of biomolecules**

**Supplementary Method 5. Investigation of the cellular BTP uptake**

**Supplementary Method 6. Co-localisation imaging**

**Supplementary Method 7. Photoluminescence of BTP in the presence of bicelles**

**Supplementary Method 8. Lipid oxidation analysis using UPLC-MS**

**Supplementary Method 9. Ca^2+^ and K^+^ mobilisation**

**Supplementary Method 10. Live-SIM imaging of mitochondrial morphology change**

**Supplementary Method 11. Mitochondrial depolarisation assay**

**Supplementary Method 12. Imaging for cell death morphology change**

**Supplementary Method 13. Cell viability assay**

**Supplementary Fig. 1.** Synthetic pathway of BTP.

**Supplementary Fig. 2-9.** ^1^H NMR, ^13^C NMR, HRMS, and FT-IR spectra for compound 3 and BTP.

**Supplementary Fig. 10.** Photophysical properties of BTP.

**Supplementary Fig. 11.** Proposed scheme of electron transfer for reductive and oxidative quenching cycles.

**Supplementary Fig. 12.** Time-correlated single-photon counting (TCSPC) spectra of BTP.

**Supplementary Fig. 13.** ∙OH generation assay using HPF.

**Supplementary Fig. 14.** Electrochemical photooxidation of amino acids.

**Supplementary Fig. 15.** ^1^O_2_ generation assay using ABDA.

**Supplementary Fig. 16.** Methionine oxidation by BTP photocatalysis.

**Supplementary Fig. 17.** BTP uptake at 37 ℃, at 4 ℃, and in the presence of NaN_3_.

**Supplementary Fig. 18.** Co-localisation of BTP in HeLa cells.

**Supplementary Fig. 19.** Co-localisation of BTP in A549 and PANC-1 cells.

**Supplementary Fig. 20.** Changes in location of BTP after photocatalysis.

**Supplementary Fig. 21.** Live-SIM images using HeLa cells before and after BTP photocatalysis.

**Supplementary Fig. 22.** Co-localisation of BTP with a plasma membrane staining dye.

**Supplementary Fig. 23.** Intracellular membrane localisation of BTP and intracellular ROS generation.

**Supplementary Fig. 24.** H_2_DCF-DA assay for intracellular ROS generation in HeLa cells.

**Supplementary Fig. 25.** H_2_DCF-DA assay for intracellular ROS generation in A549 and PANC-1 cells.

**Supplementary Fig. 26.** O_2_^−∙^ assay using dihydroethidium.

**Supplementary Fig. 27.** Lipid oxidation analysis using ultra-performance liquid chromatography-MS.

**Supplementary Fig. 28.** Single-molecule forced-unfolding assay for control conditions.

**Supplementary Fig. 29.** Volcano plots for oxidative modifications of each amino acid.

**Supplementary Fig. 30.** Gating strategies for flow cytometry experiments.

**Supplementary Fig. 31.** Pyroptotic morphology changes of A549 and PANC-1 cells.

**Supplementary Fig. 32.** Cell death modality induced by BTP photocatalysis.

**Supplementary Fig. 33.** GSDMD expression level of GSDMD knocked-out or wild-type iBMDM.

**Supplementary Fig. 34.** Western blot analysis of A549 and PANC-1 cells for caspase-4/5 cleavage.

**Supplementary Fig. 35.** Proposed mechanism of non-canonical pyroptosis induced by photocatalytic membrane oxidation.

**Supplementary Table 1.** Modifications considered during the second search to identify oxidized amino acids.

**Supplementary Method 1. Synthesis.** All chemicals used in organic synthesis were purchased from Sigma Aldrich, Alfa Aesar, Tokyo Chemical Industry, Combi-Blocks, and SAMCHUN. In the synthetic process, the newly synthesised chemical compounds were analysed by ^1^H, ^13^C NMR (Agilent 400MR-DD2 NMR spectroscopy), FT-IR (Varian Cary 620/670 FT-IR spectrometer), and HRMS (Bruker maXis^TM^ HD Ultra-high-resolution Q-TOF LC-MS/MS system, the Cooperative Laboratory Center of Pukyong National University, Republic of Korea). The synthetic scheme of BTP was referred previously reported paper[^1^](#_ENREF_1).

- 1. **Synthesis of 4-bromo-*N,N*-diphenylaniline (1)**.

A round bottom flask was filled with triphenylamine (1.0 g, 4.1 mmol) and degassed anhydrous DMF. The solution was put in an ice bath and stirred for 30 minutes. After 30 minutes, *N-*bromosuccinimide was added to the reaction mixture and stirred for 4 hours. After 4 hours, water was added to quench the reaction, and to precipitate a white powder. The white powder was filtered and washed with water, then dried at 70 °C. Yield: 97% (1.3 g, 4.0 mmol). ^1^H NMR (400 MHz, d^6^-DMSO): δ (ppm) = 7.42 (d, J = 8.88 Hz, 2H), 7.31 (m, 4H), 7.08 (tt, 7.4 Hz, 1.08 Hz, 2H), 7.02 (dd, 7.52 Hz, 1.12 Hz, 4H), 6.89 (d, J = 8.88 Hz, 2H). ^13^C NMR (100 MHz, d^6^-DMSO): δ (ppm) = 147.17, 132.61, 130.10, 125.04, 124.67, 124.13, 123.97 114.27.

**1-2. Synthesis of *N,N-*diphenyl-4-(4,4,5,5-tetramethyl-1,3,2-dioxaborolan-2-yl)aniline (2).**

A round bottom flask was charged with the **1** (2.5 g, 7.7 mmol) and anhydrous 100 mL THF (Ar bubbled), and the reaction mixture was cooled to −78 ℃. Then, the 2.5 M n-BuLi (5.24 mL, 13.11 mmol) was added dropwise over 5 minutes. 30 minutes after stirring, 2-Isopropoxy-4,4,5,5-tetramethyl-1,3,2-dioxaborolane (2.44 g, 13.11 mmol) was slowly added to the reaction mixture. Once the addition was complete, the reaction mixture was slowly warmed to room temperature and stirred overnight. Then, the reaction mixture was quenched by 100 mL water, and transferred to a separatory funnel with ethyl ether. The organic phase was collected and dried over MgSO_4_. The product was further purified by column chromatography with hexane/ethyl acetate (EA) (10:1, v/v). Yield: 55% (1.6 g, 4.3 mmol). ^1^H NMR (400 MHz, d^6^-DMSO): δ (ppm) = 7.54 (d, J = 8.56 Hz 1H), 7.32 (m, 4H), 7.09 (tt, J = 7.4 Hz, 1.04 Hz, 2H), 7.04 (dd, J = 7.48 Hz, 0.88 Hz, 4H), 6.89 (d, J = 8.56, 2H), 1.26 (s, 12H). ^13^C NMR (100 MHz, d^6^-DMSO): δ (ppm) = 150.59, 147.08, 136.21, 130.09, 125.35, 124.28, 121.08, 83.77, 25.08.

- 1. **Synthesis of 4-(7-bromobenzo[c][1,2,5]thiadiazol-4-yl)benzaldehyde (3).**

4,7-dibromobenzo[c][1,2,5]thiadiazole (500 mg, 1.70 mmol), (4-formylphenyl)boronic acid (170 mg, 1.13 mmol), Pd(OAc)_2_ (8 mg, 0.034 mmol), and KOAc (167 mg, 1.70mmol) were prepared in a flame dried two-neck round bound flask. Degassed N,N-dimethylacetamide was injected into the flask. The reaction temperature was alleviated at 90 ℃ for 3 days after stirring the mixture for 30 minutes. The raw materials were gained by extraction with 250 mL CH_2_Cl_2_ and the organic solvent was entirely evaporated by a rotary evaporator. Column chromatography was performed to get only yellow solid products using hexane/CH_2_Cl_2_ (1:1, v/v) eluent. Yield: 49.4% (177 mg, 0.558 mmol). ^1^H-NMR (400 MHz, CDCl_3_) δ (ppm): 10.12 (s, 1H), 8.10 (d, J = 8 Hz, 2H), 8.06 (d, J = 8 Hz, 2H), 7.97 (d, J = 4 Hz, 1H, 7.67 (d, J = 8 Hz, 1H). ^13^C-NMR (400 MHz, CDCl3) δ (ppm): 191.78, 153.86, 142.40, 136.01, 132.44, 132.15, 129.98, 129.78, 128.94, 114.64. FTIR (neat, cm^─1^): 3086, 2816, 2727, 1696, 1603, 1564, 1530, 1506, 1481, 1421, 1386, 1307, 1274, 1216, 1172, 1152, 1108, 1019, 934, 896, 862, 868, 839, 821, 785, 722, 678, 649, 618, 554, 518, 500, 465. HRMS (LC/Q-TOF) calc. for C_13_H_7_BrN_2_OS= 317.9462. Found: m/z =317.9461 [(M+H)^+^]. Mass error = 0.55 ppm.

**1-4. Synthesis of 4-(7-(4-(diphenylamino)phenyl)benzo[c][1,2,5]thiadiazol-4-yl)benzaldehyde (4).**

A round bottom flask was filled with **2** (300 mg, 0.81 mmol), **3** (180 mg, 0.56 mmol), Tris(dibenzylideneacetone)dipalladium(0) (30 mg, 0.033 mmol), 2-Dicyclohexylphosphino-2′,4′,6′-triisopropylbiphenyl (30 mg, 0.066 mmol), potassium phosphate aqueous solution (2 M) (1.8 mL, 3.24 mmol), and degassed THF. The solution was refluxed at 66 ℃ overnight. When the reaction was finished, water was added to quench the reaction. The product was extracted with DCM, and the organic layer was dried over MgSO_4_. The crude product was purified by column chromatography using hexane/DCM solution (2:1, v/v) as eluent. Yield: 84% (227 mg, 0.47 mmol). ^1^H NMR (400 MHz, d^6^-DMSO): δ (ppm) = 10.11 (s, 1H), 8.26 (d, J = 8.0 Hz, 2H), 8.08 (d, J = 4.0 Hz, 2H), 8.07 (d, J = 4.0 Hz, 1H), 7.99 (d, J = 8.0 Hz, 2H), 7.96 (d, J = 4.0, 1H) 7.37 (m, 4H) 7.12 (m, 8H). ^13^C NMR (100 MHz, d^6^-DMSO): δ (ppm) = 193.22, 153.65, 148.13, 147.24, 143.03, 135.92, 133.32, 130.70, 130.54, 130.49, 130.14, 130.09, 129.84, 127.66, 125.04, 124.11, 122.52

**1-5. Synthesis of (E)-2-cyano-3-(4-(7-(4-(diphenylamino)phenyl)benzo[c][1,2,5]thiadiazol-4-yl)phenyl) acrylic acid (BTP)**.

In a dried two neck round bottom flask, compound **4** (46 mg, 0.095 mmol), ammonium acetate (73 mg, 0.95 mmol) and cyanoacetic acid (162 mg, 1.90 mmol) were added under argon. Degassed acetic acid was used as a solvent, and then the mixture was refluxed at 120 ℃ for 16 hours. After cooling the solution to room temperature, raw materials were extracted by CH_2_Cl_2_ and washed with brine. MgSO_4_ removed some moisture in the organic layer, and the solvent was evaporated by a rotary evaporator. The purification with the remaining crude product was performed by column chromatography using CH_2_Cl_2_/MeOH (9:1, v/v), resulting bright orange solid compound Yield = 51.9% (27 mg, 0.049 mmol). ^1^H-NMR (400 MHz, CDCl_3_) δ (ppm): ^13^C-NMR (400 MHz, CDCl_3_) δ (ppm): 153.73, 153.68, 148.00, 147.24, 139.33, 133.37, 132.80, 130.81, 130.66, 130.60, 130.13, 129.95, 129.87, 129.21, 127.72, 125.00, 124.06, 122.55, 119.61. FTIR (neat, cm^−1^): 3391, 2923, 2852, 2256, 2215, 2129, 1626, 1589, 1514, 1483, 1388, 1363, 1332, 1281, 1218, 1193, 1180, 1153, 1122, 1049, 1025, 1003, 888, 824, 788, 755, 731, 696, 679, 655, 621, 590, 554, 522, 510. HRMS (LC/Q-TOF) calc. for C_33_H_23_N_4_S = 507.1638. Found: m/z = 507.1642 [(M−CO_2_+H)^+^]. Mass error = −0.79 ppm.

**Supplementary Method 2. Photophysical and electrochemical properties.**

**2-1. Absorption and photoluminescence spectroscopy.**

The aqueous solutions of BTP (20 μM, H_2_O:DMF = 99:1 v/v%) was prepared for measuring the absorbance and photoluminescence of BTP. The UV-visible spectrometer (SHIMADZU UV-2600 240V EN, Japan) and fluorescence spectrometer (ISS PC1 photon counting spectrofluorometer, USA) were utilised. The absorption and fluorescence spectra were normalised by maximum point of each spectrum in visible range. Then, we obtained the wavelength of the cross-point of absorbance and fluorescence to set E^0/0^ (525 nm, 2.36 eV).

**2-2. Photoinduced electron transfer at steady state.**

In the reductive quenching cycle, the excited BTP has proper potential to oxidise water. Thus, photoinduced electron transfer can occur between excited BTP and water molecules, reducing the fluorescence of BTP. To this end, the 20 μM BTP solution (in acetonitrile) was prepared to measure fluorescence change depending on the percentage of H_2_O. The fluorescence of BTP was measured by increasing H_2_O content from 0% to 12% at 2% interval.

**2-3. Time-correlated single photon counting (TCSPC).**

The lifetimes of time-resolved photoluminescence (PL) decays of BTP with different water concentration was measured by TCSPC (FluoTime300, PicoQuant). The sample (20 µM BTP) was dissolved in each different ratio of acetonitrile and water (%H_2_O = 0, 5, and 10). Photoexcitation of samples was operated through 450 nm ps pulse and a continuous wave diode laser head (LDH-D-C-450) with a PDL 820 laser drive. The photoluminescence from the BTP was detected by TCSPC module (PicoHarp 300E, PicoQuant) with a photomultiplier tube (PMA-C 182-N-M, PicoQuant). The decay signals and the instrument response function were fitted using fitting software (FluoFit, PicoQuant), resulting in the PL lifetime.

**Supplementary Method 3. Reactive oxygen species (ROS) generation.**

**3-1. Singlet oxygen assay (9,10-anthracenediyl-bi(methylene)dimalonic acid, ABDA).**

The singlet oxygen (^1^O_2_) generation was measured by a ^1^O_2_ indicator, 9,10-anthracenediyl-bi(methylene)dimalonic acid (ABDA) (Sigma Aldrich, USA). 100 mM stock solution of ABDA was added to each 5 µM methylene blue (MB) and BTP to 1:1000 volume ratio (20:1 molar ratio). These solutions were irradiated by LED (λ = 630 nm for MB, λ = 450 nm for BTP) for 0, 0.1, 0.2, 0.3, and 0.4 J∙cm^−2^, and the ABDA absorbance at 380 nm was measured by microplate reader. The ABDA absorbance decay of each sample was recorded as Abs_ABDA, 380nm_ − Abs_sample, 380nm_ to correct baseline.

**3-2. Intracellular ROS assay (2′,7′-Dichloro-dihydrofluorescein diacetate, H_2_DCF-DA)**

The DCFH_2_-DA is well known ROS indicator in cell environment. For intracellular ROS assay, HeLa, PANC-1, and A549 cells were grown on the cell culture dish for 24 hours. The cells (ATCC, USA) were incubated in the 5 μM BTP-containing culture media (media:DMF = 200:1, v/v) for 2 hours and then treated with the 20 μM H_2_DCF-DA in serum-free media for 40 min. After PBS washing, the cells were irradiated by the blue LED light (λ_max_ = 450 nm, 5 J∙cm^−2^), then the cells were imaged with Carl Zeiss LSM980 laser-scanning confocal microscopy (Jena, Germany). The 488 nm-laser and 20X objective lens are used to detect DCF. The Emission gain was (500-530 nm) to remove fluorescence of BTP. Three negative controls (BTP+/light−, BTP−/light+, and BTP−/light−) was involved in this experiment, and the cells were imaged with Carl Zeiss LSM980 in the CO_2_ incubator at 37 °C under humidified atmosphere and 5% CO_2_. Further, the DCF fluorescence of randomly selected 20 individual cells before and after BTP photoactivation was described as dot plot.

**3-3. Superoxide radical assay (Dihydroethidium, DHE)**

The DHE is a selective superoxide radical indicator, enhancing its fluorescence by intracellular superoxide radicals. The DHE molecules are oxidised by the superoxide radical, then form longer conjugation system. The oxidised form of DHE is intercalate DNA or RNA of cells (nuclear and mitochondrial DNA), emitting strong fluorescence. Thus, HeLa cells were grown on the cell culture dish for the DHE assay, and the cells were treated to 10 μM BTP-containing media for 2 hours. After washing with PBS several times, the cells were further incubated in the 5 μM DHE-containing media in serum-free media. Then, the HeLa cells were irradiated by the blue LED light (λ_max_ = 450 nm, 10 J∙cm^−2^), and imaged with confocal microscopy (Carl Zeiss LSM980). The BTP+/light− condition was also involved in this experiment as negative control, and the cells were imaged with Carl Zeiss LSM980 in the CO_2_ incubator at 37 ℃ under humidified atmosphere and 5% CO_2_.

**Supplementary Method 4. Photocatalytic oxidation of biomolecules**

**4-1. Photocatalytic amino acid oxidation in oxidative quenching cycle.**

For fabrication of the working electrode (BTP@TiO_2_/FTO), a fluorine-doped tin oxide (FTO, Nippon Sheet Glass Co., Ltd) substrate was cleaned-up with ultrasonication using acetone, ethanol, and D.I. water sequentially for 10 min each. Mesoporous TiO_2_ layer was prepared by screen-printing a TiO_2_ paste (30NR-D, Greatcell Solar Materials Pty. Ltd.) on the FTO with a printing area of 1.0 × 0.7 cm^2^. The TiO_2_-printed substrate was gradually heated at 150 ℃ for 10 min, at 325 ℃ for 5 min, at 327 ℃ for 5 min, at 450 ℃ for 15 min, and at 500 ℃ for 30 min. The TiO_2_/FTO was immersed in 0.2 mM solution of BTP in chloroform/ethanol (7:3; v/v) for 18 h at room temperature, under dark condition. After the soaking process, the substrate was washed by acetonitrile to remove unadsorbed BTP molecules, giving the BTP-adsorbed working electrode (BTP@TiO_2_/FTO). Chronoamperometry measurements were carried out using Vertex Potentiostat/Galvanostat (IVIUM technologies, Eindhoven, Netherlands) equipped with a standard three-electrode system, consisting of the BTP@TiO_2_/FTO as the working electrode, a Pt wire as the counter electrode, and an Ag/AgCl (saturated KCl solution) as the reference electrode. To observe photocatalytic redox reaction of BTP with amino acids, 13 types of electrolytes containing each amino acid (L-tryptophane, L-alanine, arginine·HCl, tyrosine, serine, glutamine, histidine, phenylalanine, proline, lysine·HCl, glutamic acid, methionine, and cysteine) were prepared (0.05 mmol of amino acid in 20 mL degassed 1× PBS). In a glass chamber, all the electrodes connected to the instrument were positioned as shown in the figure below, in which especially the FTO side of the working electrode should face the light source. With an applied potential of 0.0 V vs. NHE (−0.197 V vs. Ag/AgCl), the light on/off chronoamperometry measurements were recorded for each amino acid electrolyte (10 s light-on and 10 s light-off, 450 nm blue LED). The corresponding photocurrent generation under the light-on indicates the amount and rate of amino acid oxidation by the photoexcited BTP molecules.


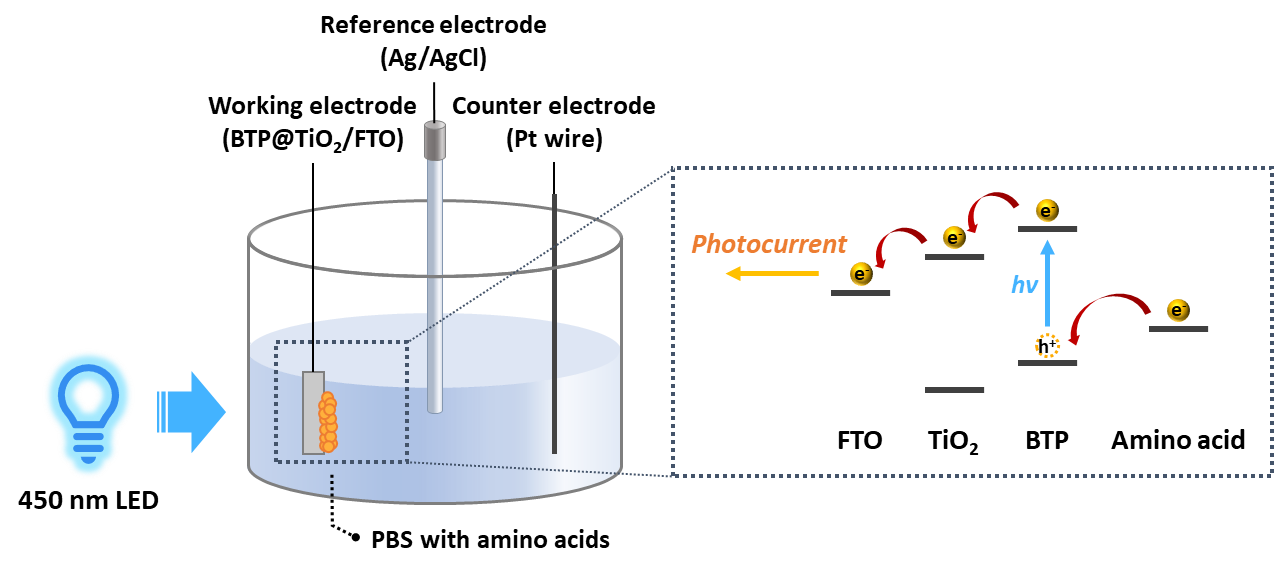


**4-2. HRMS for methionine oxidation product.**

Methionine is known to be easily oxidised by ROS such as singlet oxygen, superoxide radical, and hydroxyl radical. Thus, methionine oxidation by the BTP photoactivation was confirmed by the high-resolution mass spectroscopy (HRMS) (maxis HD, Bruker). The experimental solution (0.1 mM BTP and 1 mM methionine in PBS:DMF = 99:1, v/v) was prepared to oxidise methionine. The solution was irradiated by the blue LED light (λ_max_ = 450 nm, 16.6 mW∙cm^−2^ for 2 hours). Then, all solvent was evaporated, and dried by Ar gas to obtain powder sample. The sample was stored at 4 ℃ under light-shielding condition before injection to the HRMS. The dried sample was eluted with H_2_O, the m/z value was measured by the electrospray ionisation (ESI) in the positive mode. The capillary voltage was 4500 V; charging voltage was 2000 V; dry gas temperature was 200 ℃; and mass range was 50-1000 m/z. The peak with m/z = 166.0532 were assigned as the oxidised methionine (calculated m/z value of [O-Met + H]^+^ is 166.0532). This oxidised methionine peak was not found from the negative control experiment (BTP+/hv−).

**Supplementary Method 5. Investigation of the cellular BTP uptake.**

To investigate how cells uptake BTP molecules, intracellular BTP fluorescence under physiological conditions at 37 ℃, at 4 ℃, and in the presence of NaN_3_ was examined using confocal microscopy. HeLa cells were pre-incubated under three conditions (37 ℃, at 4 ℃, and in the presence of 100 mM NaN_3_) for 30 minutes, and then further incubated with BTP (10 μM) for 2 hours. Afterward, the intracellular BTP fluorescence was measured using the laser-scanning confocal microscope LSM980 (Carl Zeiss, Germany).

**Supplementary Method 6. Co-localisation imaging.**

The confocal laser scanning microscopy (CLSM) was utilised to identify subcellular localisation of BTP. One or two days prior to the experiments, HeLa, A549, and PANC-1 cells were seeded on coverglass-bottom confocal dish. The cells were transfected by Sec61b-mGFP construct and Turbofect® transfection reagent (Thermo Fisher, USA) to stain endoplasmic reticulum (ER) and by Cell Light™ Golgi RFP BacMam2.0 (Thermo Fisher, USA) to stain Golgi apparatus. One day after transfection, the cells were incubated with BTP (5 μM in media:DMF = 200:1, v/v) for 2 hours. Then, the co-localisation images were obtained in the CO_2_ incubator at 37 ℃ under humidified atmosphere and 5% CO_2_ by Carl Zeiss LSM980 with 63X lens. (Sec61b-mGFP: λ_excitation_ = 488 nm, emission gain range: 500-530 nm; Cell Light™ Golgi RFP BacMam2.0: λ_excitation_ = 561 nm, emission gain range: 534-630 nm; BTP: λ_excitation_ = 445 nm, emission gain range: 550-620 nm). For co-localisation imaging with mitochondria, the grown HeLa, A549, and PANC-1 cells were incubated with BTP (5 μM in media:DMF = 200:1, v/v) for 2 hours. After the incubation with BTP, the cells were stained by the 500 nM MitoTracker^TM^ Deep Red FM (Invitrogen, M22426). Then, the cells were imaged with Carl Zeiss LSM980 in the CO_2_ incubator. (MitoTracker^TM^ Deep Red FM: λ_excitation_ = 647 nm, emission gain range: 650-700 nm; BTP: λ_excitation_ = 445 nm, emission gain range: 550-620 nm). To monitor the location changes of BTP by photoactivation, the cells stained by BTP and MitoTracker^TM^ Deep Red FM were irradiated by the 445 nm confocal laser (4%) for 5 min. Further, the time-series CLSM monitored the change in localisation of BTP for 644.4s (50 frames). To support the location change, live structured illumination microscopy (Live-SIM) was used (Carl Zeiss ELYRA S. 1, Germany). For Live-SIM, HeLa cells were incubated with BTP (10 μM in media:DMF = 100:1, v/v) for 2 hours, and then further incubated in culture media containing MitoTracker^TM^ Deep Red FM. In the CO_2_ incubator, 488 nm laser and 642 nm laser with 63X lens were used to excite BTP and MitoTracker^TM^ Deep Red FM, respectively. To calculate single Live-SIM images, 5 slices of parallel images was used (109.55 nm interval for the z-axis). BTP was photoactivated by the 488 laser (5%) of ELYRA S. 1, then location change of BTP was imaged by Live-SIM in the same method. Additionally, the location changes to the plasma membrane after photocatalysis were investigated using CellMask™ plasma membrane stains Deep Red. HeLa cells were incubated with BTP (5 μM in media:DMF = 200:1, v/v) for 2 hours. Subsequently, the cells were stained with the CellMask™ plasma membrane stains Deep Red 10 minutes before imaging. The colocalisation images were obtained both before and after photoactivation using the 445 nm confocal laser (4%) for 5 minutes.

**Supplementary Method 7. Photoluminescence of BTP in the presence of bicelles.**

Aqueous solutions of BTP (20 μM) in pH 7.5 Tris buffer (50 mM Tris and 150 mM NaCl) were prepared for measuring the photoluminescence of BTP. The fluorescence spectra were measured using a microplate reader (λ_ex_ = 450 nm; SpectraMax M5e). Subsequently, the photoluminescence of BTP in the presence of bicelles was also measured in 50 mM Tris (pH 7.5), 150 mM NaCl, and 2.0% bicelle. Lipid bilayer discs (bicelles) consist of DMPC lipid and CHAPSO detergent at a 2.5:1 molar ratio. Furthermore, Lambda-scan microscopy (LSM980) was used to measure the emission peak of intracellular BTP. HeLa cells were incubated with BTP (10 μM) for 2 hours, then imaged using a confocal microscopy. Using 445 nm laser, 22 images of intracellular BTP were imaged at each wavelength from 468 nm to 689 nm (at 8.5 nm interval). The averaged emission intensities of each image were calculated and then plotted as a function of wavelength.

**Supplementary Method 8.** **Lipid oxidation analysis using UPLC-MS.**

To analyse lipid oxidation induced by BTP photocatalysis, samples were prepared from two groups for comparison. (1) hv−/BTP−: the cells were cultured with neither light nor BTP treatment. (2) hv+/BTP+: the cells were incubated with BTP and exposed to 450 nm LED (λ_max_ = 450 nm, 16.67 mW·cm^−2^ for 10 minutes, 10 J·cm^−2^). HeLa cells were grown in a 100 mm cell culture dish with DMEM supplemented with FBS and antibiotics at 37 ℃ in a humified atmosphere containing 5% CO_2_ environment. For hv+/BTP+ conditions, the cultured cells were incubated with 10 µM BTP for 2 h, and the culture medium was exchanged with fresh DMEM before light irradiation. The cells both of hv+/BTP+ and hv−/BTP− conditions were washed with 1 mL of DPBS and collected using a cell scraper. Afterward, 2 mL of MeOH was added to the collected cells, then 0.9 mL of dichloromethane was also added to the cells. After vortex, 1 mL of water was added, and then further 0.9 mL of dichloromethane was added. Subsequently, each tube was inverted 10 times. The solution was centrifuged at 3000 × g for 10 min, and the lower layers of each tube were collected. This procedure from the addition of water was repeated one more, and the lower layers of each tube were added to first extract. The extract solutions were evaporated under a stream of nitrogen and diluted with 100 µL of isopropanol/acetonitrile/water (2:1:1) to inject ultra-high performance liquid chromatography (UPLC) system (ACQUITY I CLASS, WATERS/Q-TOF). ACQUITY UPLC CSH C_18_ column (2.1 × 100, 1.7 μm) was used. After UPLC system, the sample was injected to the MS (maxis HD, Bruker), the m/z value was measured by the electrospray ionisation (ESI) in the positive and negative mode. The capillary voltage was 2000 V for ESI+ mode and 1000 V for ESI− mode; charging voltage was 2000 V; dry gas temperature was 550 ℃; and mass range was 100-2000 m/z. This experiment was duplicated.

All RAW files from the UPLC-MS results were converted to peak lists using MSConvert (ver. 3.0.20279; http://proteowizard.sourceforge.net). The identification of 15:0-18:1 phosphatidylcholine (PC) was performed using heavy labelled (deuterated) internal lipid standards (SPLASH LipidoMIX^TM^ Internal Standard). The extracted ion chromatograms (XICs) of lipid standards were compared with the XICs of their (light) counterparts. A lipid was deemed identified in the sample if two XICs of the lipid and its heavy standard overlapped for at least three consecutive scans. Only peaks with intensities greater than 5% of the highest peak in an MS1 spectrum were taken into account, and a mass tolerance of 0.02 Da was used to match spectral peaks. For identified (non-oxidized) lipids, we calculated XICs for their oxidized forms (+13.979, +15.995, +31.99). The oxidation level of lipid was calculated as follows:

$$\frac{\sum_{\{+13.979, +15.995, +31.99\}} XIC peak area (lipid)}{\sum_{\{non-oxidized, +13.979, +15.995, +31.99\}} XIC peak area (lipid)}$$

**Supplementary Method 9. Ca^2+^ and K^+^ mobilisation.**

**9-1. Time series imaging of mitochondrial Ca^2+^ uptake.**

HeLa cells were seeded on the imaging dishes one day prior to the experiment. The cells were incubated in the culture media containing BTP (5 μM in media:DMF = 200:1, v/v) for 2 hours, and loaded with the 500 nM of MitoTracker^TM^ Deep Red FM and the 3 μM of the mitochondrial Ca^2+^ indicator, Rhod-2 (Invitrogen R1244, USA). Then the Ca^2+^ mobilisation was measured by the time-series confocal microscopy with Carl Zeiss LSM980 in the CO_2_ incubator at 37 ℃ under humidified atmosphere and 5% CO_2_. The Rhod-2 was excited by the 561 nm laser, and BTP was excited by the 445 nm laser (0.3 mW). BTP was photo-activated by the 445 nm confocal laser for 60 seconds, during the irradiation, the fluorescence signals of MitoTracker and Rhod-2 were imaged at 10 seconds intervals by 63X lens. To confirm the Ca^2+^ mobilisation depending on irradiation time, the line-cut fluorescence profile was obtained from the co-localisation images at t = 0, 30, and 60 s.

**9-2. Flow cytometry for Ca^2+^ and K^+^ mobilisation**

HeLa cells of 7–80% confluence in 6-well plate were treated with BTP (10 μM) for 2 h and further incubated with Rhod-2 AM (3 μM) and Ion K+ green-2 (40 μM) for an hour. Then, the cells were irradiated with blue LED (450 nm, 10 J∙cm^−2^). 2 hours after irradiation, the cells were detached using cell scrapper and the fluorescence of cell suspension was detected using flow cytometry (CytoFLEX S, Beckman, USA). We chose cells with positive FSC and positive SSC, and discarded cells with extremely low signal of FSC and SSC. The boundaries of FSC and SSC were 2 × 10^6^ and 1.5 × 10^6^, respectively (Supplementary Fig. 30). The fluorescence of both Rhod-2 and Ion K+ green-2 dyes were analyzed at λ_ex_=561 nm and λ_em_=610 nm. The FACS data was processed by CytExpert software (ver. 2.4.0.28).

**Supplementary Method 10. Live-SIM imaging of mitochondrial morphology change.**

Mitochondrial matrix swelling and morphological change by BTP photoactivation were imaged with the Live-SIM (Carl Zeiss ELYRA S. 1, Germany). Prior to the experiment, HeLa cells were transfected with sec61b-mCherry construct and Turbofect® transfection reagent (Thermo Fisher, USA) for staining endoplasmic reticulum (ER). The cells were incubated with BTP containing culture media (10 μM in media:DMF = 100:1, v/v), and further treated with 500 nM MitoTracker^TM^ Deep Red FM for 30 minutes. Then, the cells were irradiated (10 mW) by 488 nm laser of ELYRA S. 1 (Carl Zeiss, Germany) and imaged with 63X lens. The 488 (10%), 561 (10%), and 647 nm (1%) laser of ELYRA S. 1 were used to excite BTP, sec61b-mCherry, and MitoTracker^TM^ Deep Red, respectively. During imaging, the cells were in the CO_2_ incubator at 37 ℃ under humidified atmosphere and 5% CO_2._ To calculate single Live-SIM images, 5 slices of parallel images was used.

**Supplementary Method 11. Mitochondrial depolarisation assay.**

To estimate mitochondrial damage induced by BTP photocatalysis, mitochondrial depolarisation was investigated using the tetramethylrhodamine ethyl ester (TMRE) assay. HeLa cells were grown on a cell culture dish for 24 hours. Afterward, the cells were incubated with BTP (5 μM in media:DMF = 200:1, v/v) for 2 hours, followed by irradiation with blue LED light (λ_max_ = 450 nm, 10 J∙cm^−2^) after several washing steps with PBS. Subsequently, the cells were incubated with TMRE (500 nM) for 20 minutes and imaged using Carl Zeiss LSM980 laser-scanning confocal microscopy (Jena, Germany). Three negative controls were included in this experiment: BTP+/light−, BTP−/light+, and BTP−/light−. The cells were imaged with Carl Zeiss LSM980 inside a CO_2_ incubator at 37 ℃ in a humidified atmosphere with 5% CO_2_. Additionally, the TMRE fluorescence of 20 randomly selected individual cells was quantified as a dot plot both before and after BTP photoactivation.

**Supplementary Method 12. Imaging for cell death morphology change.**

Pyroptotic morphology change of cells was imaged with Carl Zeiss LSM780 using 63X objective lens. The HeLa, A549, and PANC-1 cells were grown on confocal dishes one day prior to the experiment. The cells were incubated with BTP (5 μM in media:DMF = 100:1, v/v) for 2 hours, and washed several times with PBS. Then the cells were imaged with LSM780 at 37 °C under humidified atmosphere and 5% CO_2_. The blue light from 445 nm laser of LSM780 was exposed to the cells to activate BTP, and morphology of the cells before and after BTP photoactivation were imaged respectively.

To investigate cell death modality, the morphological change of dying cells treated with Liproxstatin-1 (Sigma Aldrich SML1414, USA) and z-VAD-fmk (Promega G7231, USA) were imaged. The HeLa cells were seeded on the four confocal dishes. One day after seeding, the cells were incubated with a total of four conditions, w/ and w/o Liproxstatin-1 (10 µM) and z-VAD-fmk (4 µM), for 16 hours. Then, the cells were further treated to BTP (8 μM) for 2 hours and propidium iodide (1.5 μM) (Invitrogen P3566, USA) for 30 minutes. After all incubation, the cells were exposed to LED light (λ_max_ = 450 nm, 3 J∙cm^−2^) imaged with 20X lens of LSM780 at 37 ℃ under humidified atmosphere and 5% CO_2_. The Propidium iodide were excited by 561 nm laser. Further, the morphology changes of WT and GSDMD^−/−^ iBMDM by BTP photocatalysis were imaged. The WT and GSDMD^−/−^ iBMDM were grown on the confocal dishes, then incubated with BTP (8 μM) for 2 hours. After incubation, the media was changed, and the cells were exposed to LED light (λ_max_ = 450 nm, 10 J∙cm^−2^). 2 hours after the irradiation, the cells were imaged with 20X lens of LSM780 at 37 ℃ under humidified atmosphere and 5% CO_2_.

**Supplementary Method 13. Cell viability assay.**

**13-1. Live or Dead assay**

HeLa cells were grown on the confocal imaging dish one day prior to the experiment. The cells were treated with BTP (10 μM) for 2 hours, then the cells were irradiated by the blue LED (λ_max_ = 450 nm, 3.7 J∙cm^−2^). One day after irradiation, the cells were incubated with 2.5 μM of Calcein AM (Invitrogen, USA) and 1.5 μM propidium iodide (Invitrogen, USA) for 30 minutes. The LSM980 confocal microscopy (Carl Zeiss, Germany) with 20X lens obtained the signals of calcein AM and propidium iodide. The Calcein AM signal were measured by 488 laser and 490-530 nm emission gain to remove green fluorescence of BTP. The experiment was repeated on the negative controls (without BTP and/or light exposure).

**13-2. MTT assay**

HeLa cells (1.5×10^5^ cells∙mL^−1^) were seeded into the 96 well plate and incubated for overnight at 37 ℃ in the humidified CO_2_ incubator. The cells were treated with BTP (0, 2, 4, 8, 16, and 32 μM, 1% DMF) for additional 2 h and washed with phenol red-free DMEM (containing 10% foetal bovine serum, 50 units/mL penicillin, and 50 μg/mL streptomycin), followed by photoirradiation (5 J∙cm^−2^, 450 nm LED). The cells further incubated for 24 h. Subsequently, 3-(4,5-dimethylthiazol-2-yl)-2,5-diphenyltetrazolium bromide (MTT, 25 μL, 5 mg∙mL^−1^ in PBS) was treated for 3 h and the culture media were replaced with DMSO and shaken for 2 h. The absorbance of Formazan was detected using microplate reader (λ_abs_ = 570 nm, SpectraMax M5e). For pancreatic cancer cells, PANC-1 and Mia paca-2 (2×10^4^ cells∙well^−1^) were seeded on 96-well plate in hypoxic or normoxic conditions. After overnight allowed to adhere, the cells were treated 0 to 32 μM BTP for 2 h. Changed to culture media, cells were exposed to 450 nm irradiation(5 J∙cm^−2^) and further incubated for 24 h. MTT reagent (MTT-CAS 298-93-1–Calbiochem, Sigma-Aldrich, Cat.No. 475989, USA) was added to 25 μL each well (Final concentration 1mg∙ml^−1^), and the plates were incubated at 37℃ for 4 h. Following incubation, dissolution of the resulting formazan in 100 μL of DMSO. Then, absorbance was recorded at 570 nm using Infinite MPlex plate reader (Tecan, Mannedorf, Switzerland). Before seeding, cells were cultured under hypoxic (1% O_2_, 5% CO_2_, and 37℃) and normoxic condition(20% O_2_, 5% CO_2_, and 37℃) at least cells were cultured two times on those conditions.

**Supplementary Figures**

**
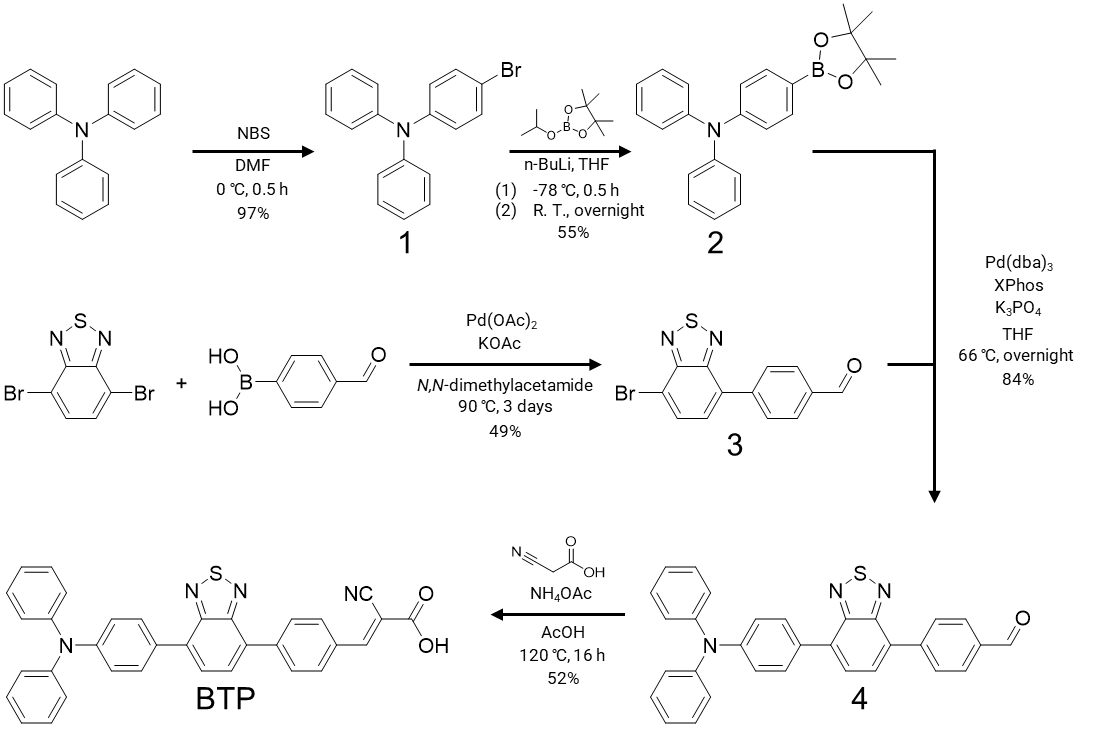
**

**Supplementary Fig 1.** Synthetic pathway of BTP


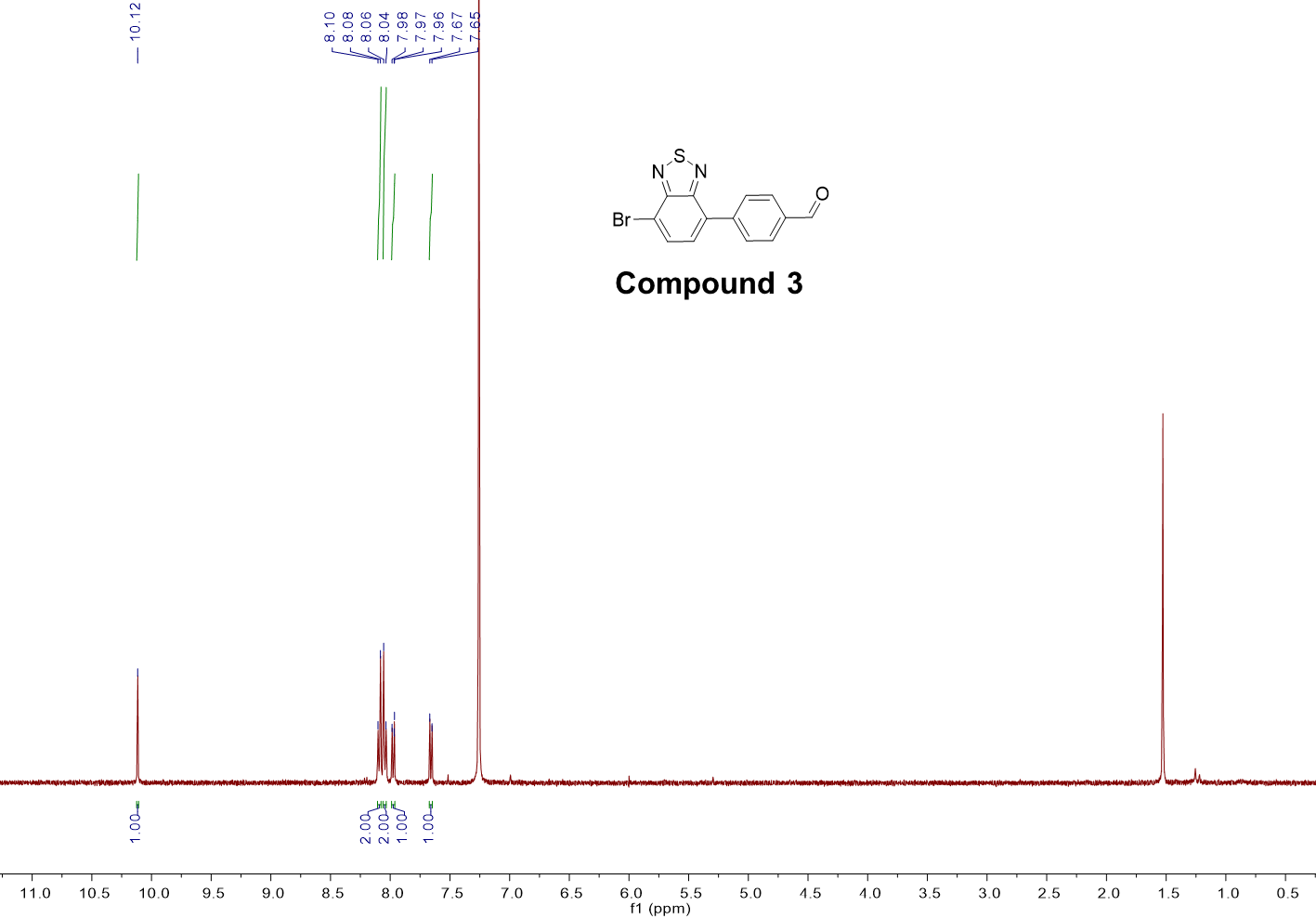


**Supplementary Fig. 2.** ^1^H-NMR of compound 3 (CDCl_3_, 400 MHz, 298 K)

**
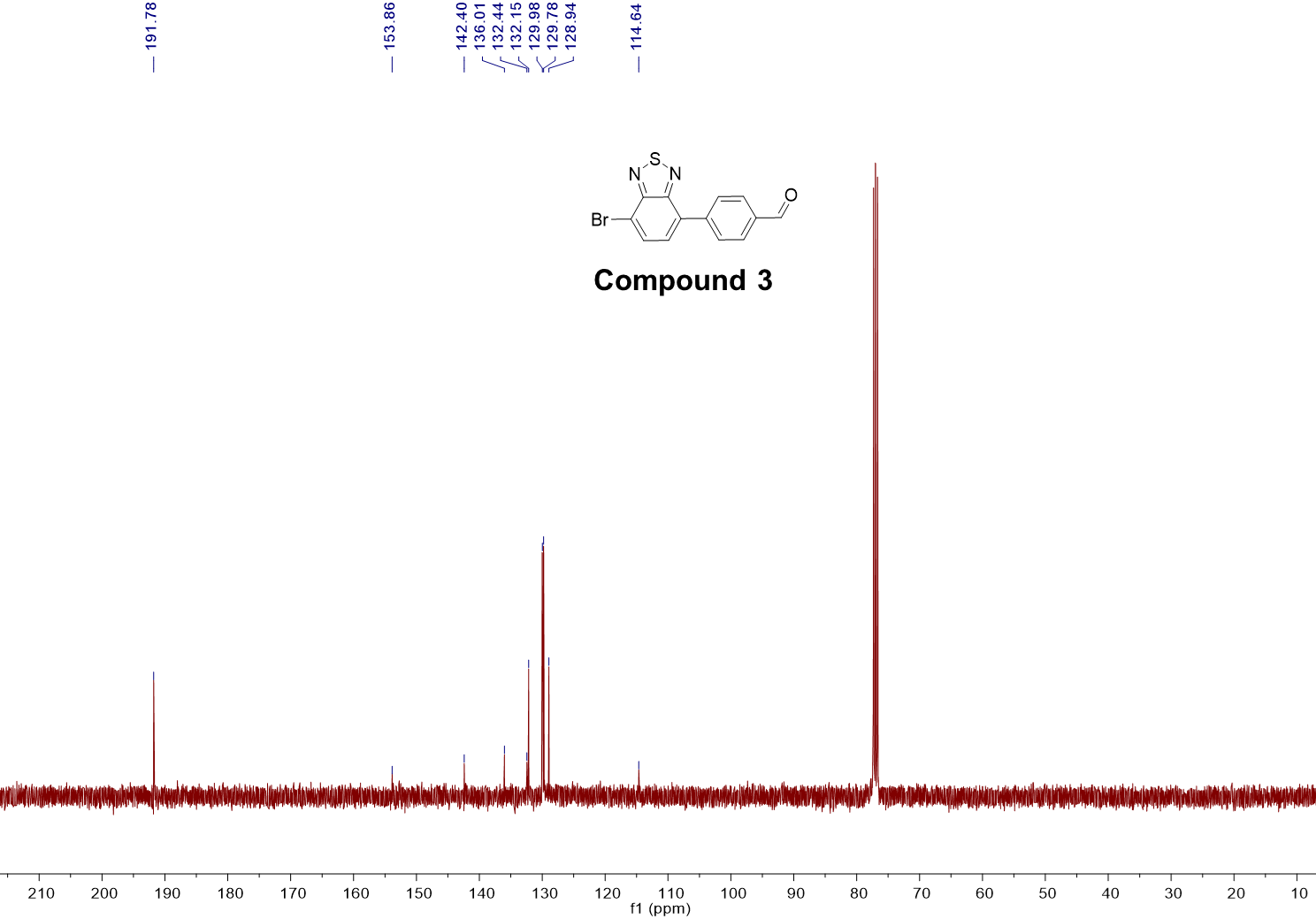
**

**Supplementary Fig. 3.** ^13^C-NMR of compound 3 (CDCl_3_, 400 MHz, 298 K)


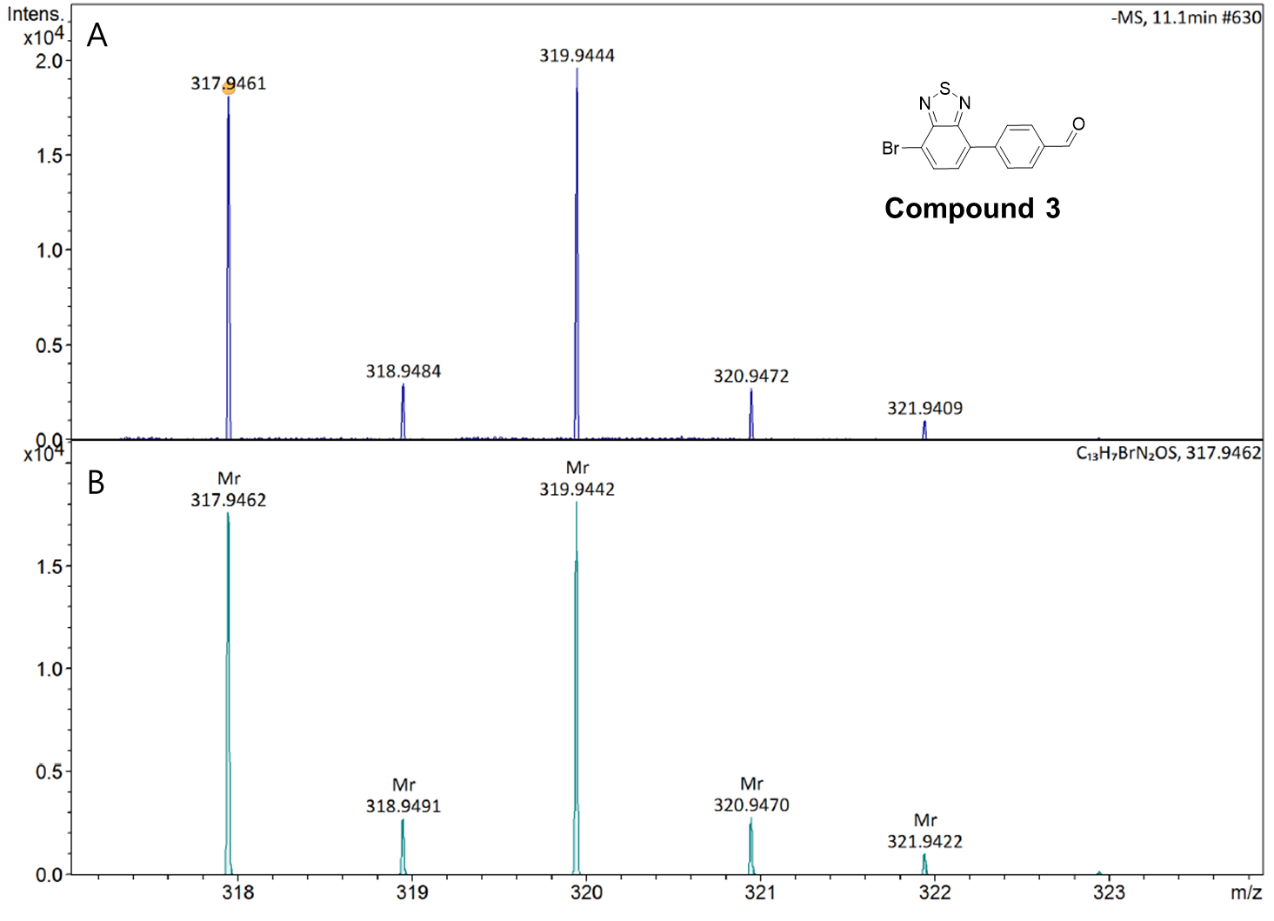


**Supplementary Fig. 4.** (A) Experimental (B) simulated HRMS spectra of compound 3.


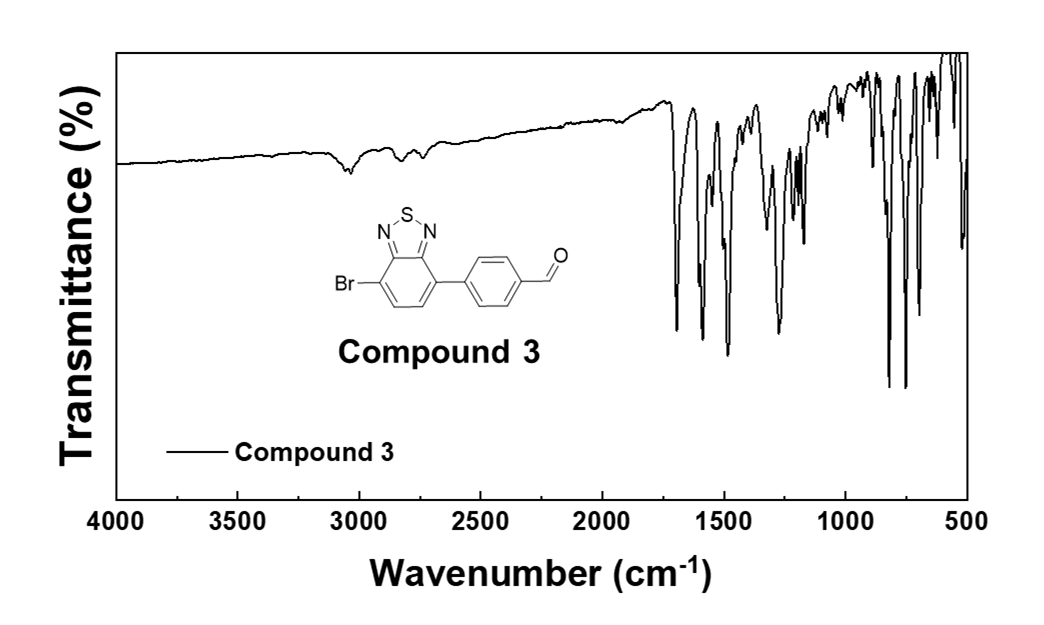


**Supplementary Fig. 5.** FT-IR of compound 3


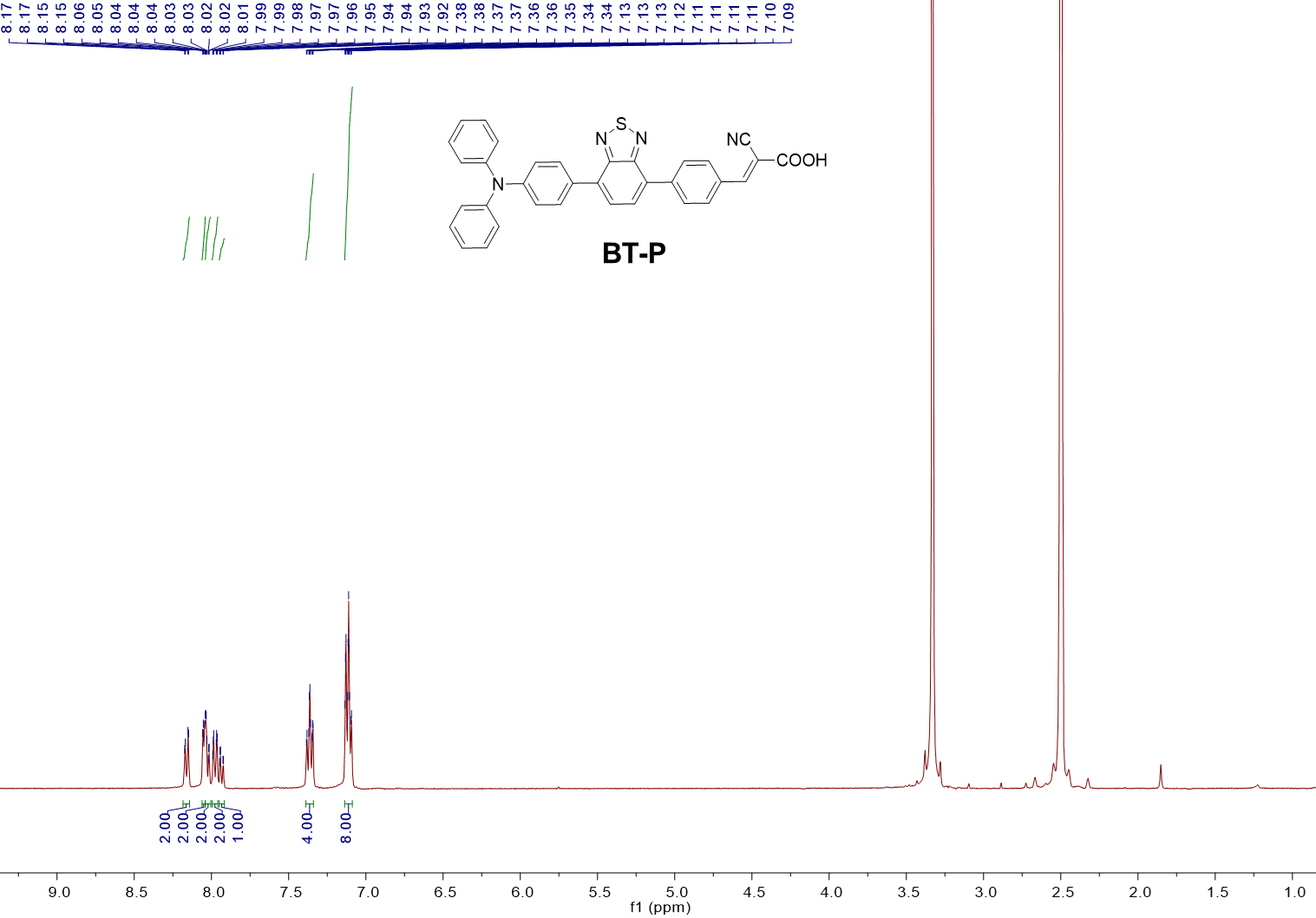


**Supplementary Fig. 6.** ^1^H-NMR of compound BTP (CDCl_3_:DMSO = 1:1, 400 MHz, 298 K)


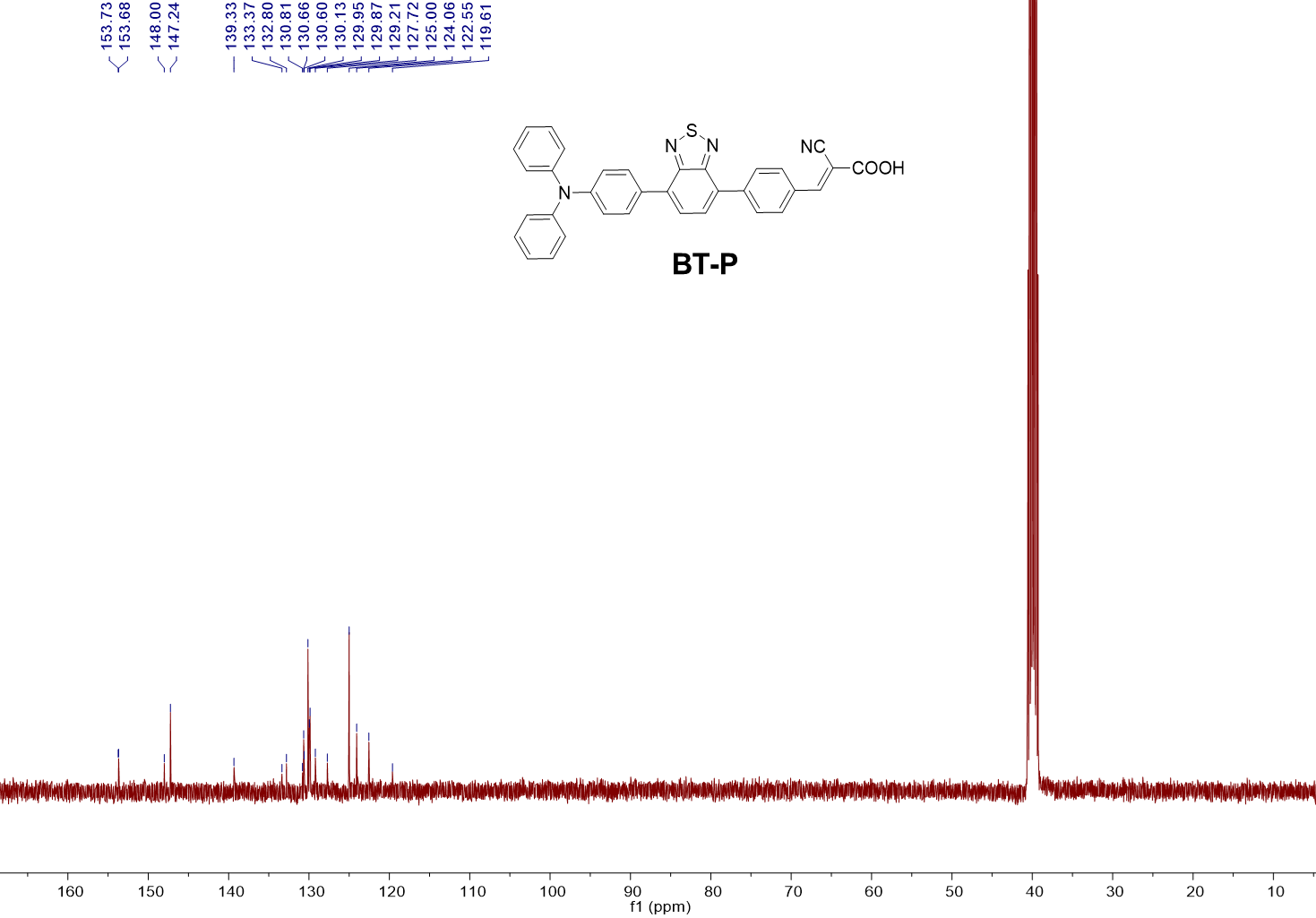


**Supplementary Fig. 7.** ^13^C-NMR of compound BTP (CDCl_3_:DMSO = 1:1, 400 MHz, 298 K)


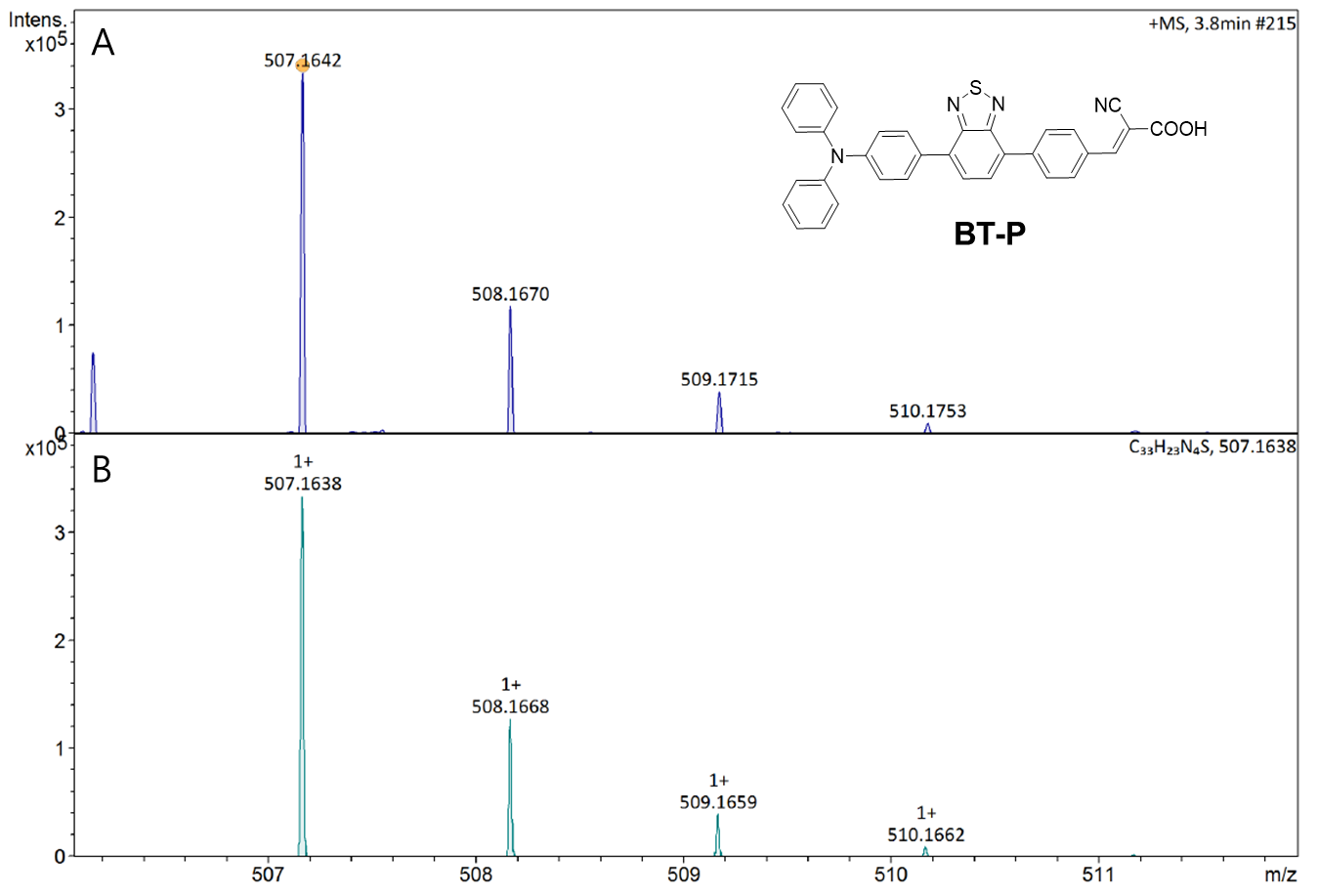


**Supplementary Fig. 8.** (A) Experimental (B) simulated HRMS spectra of BTP.


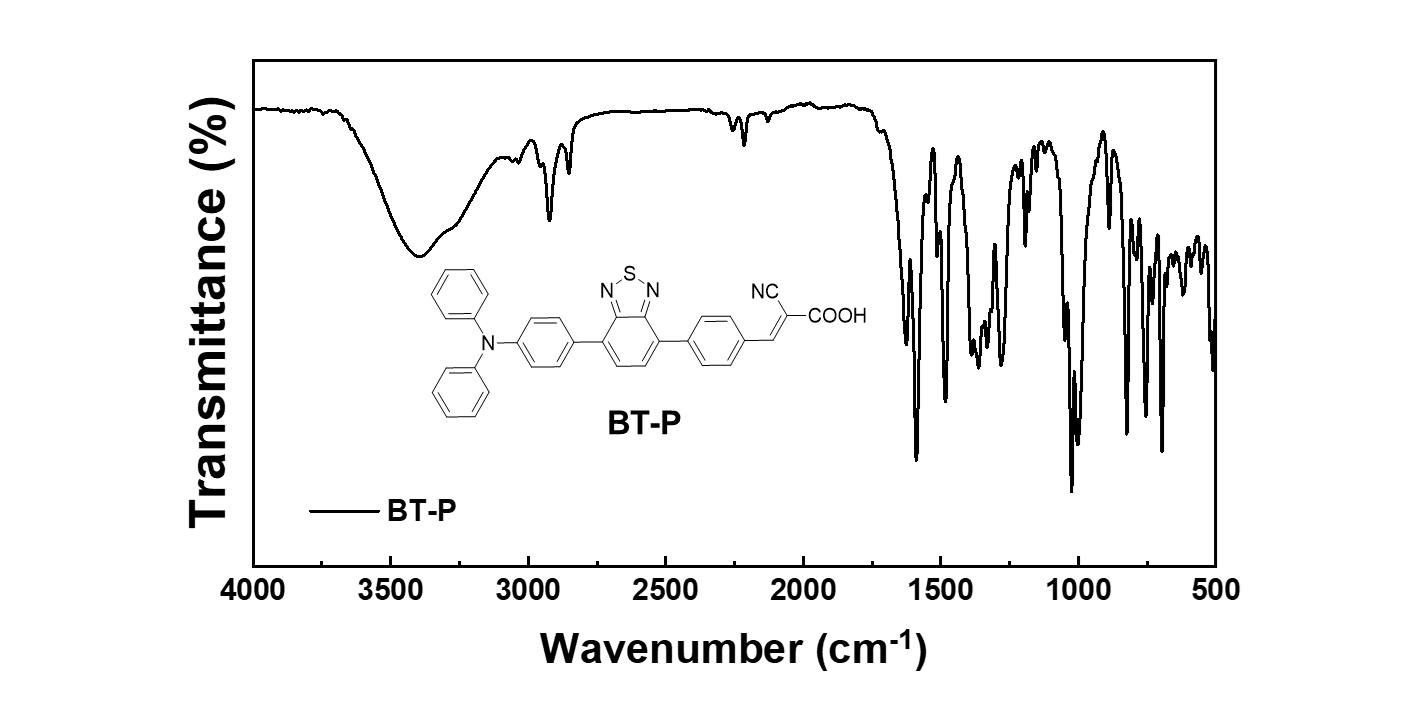


**Supplementary Fig. 9.** FT-IR of compound BTP


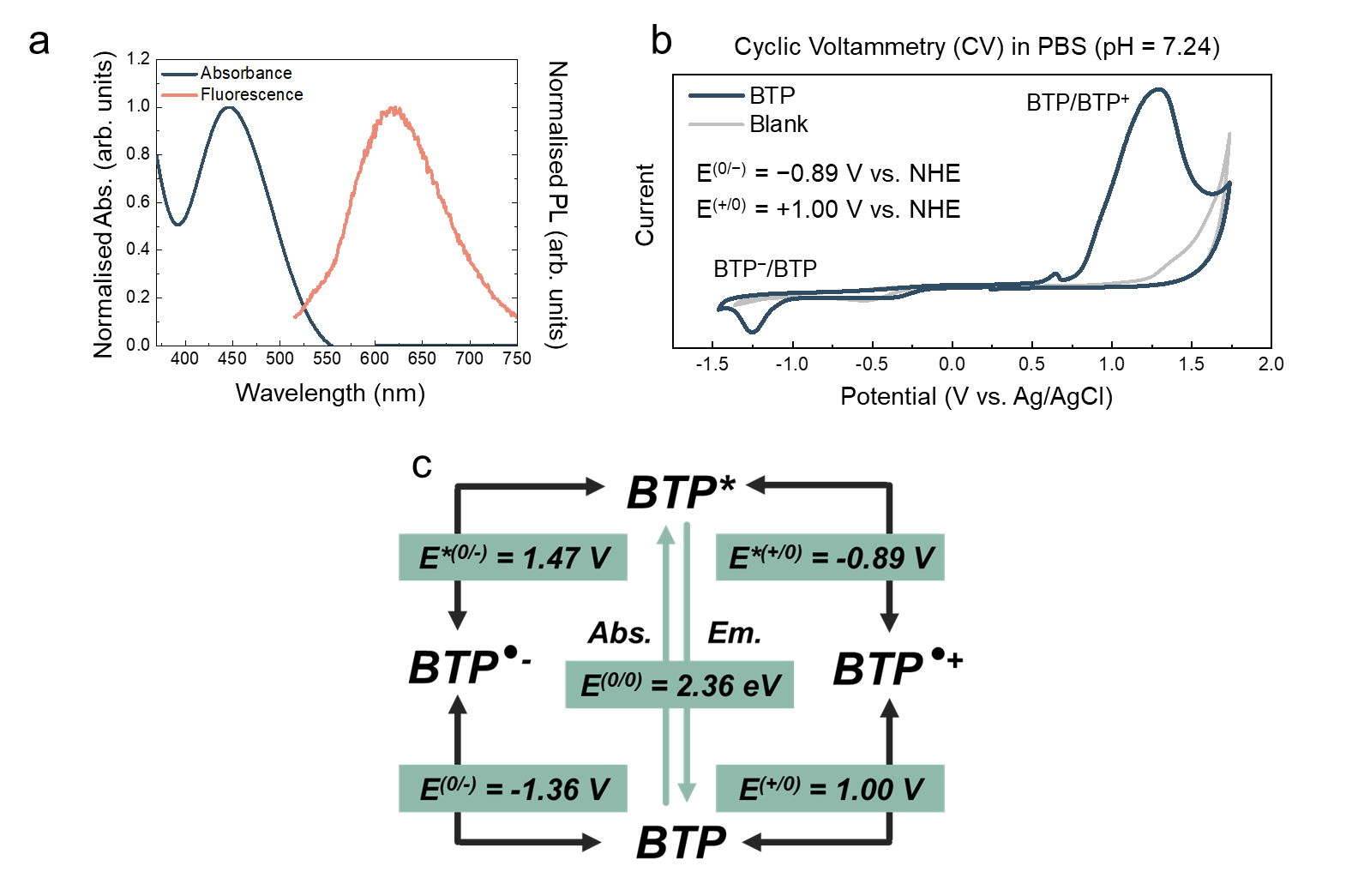


**Supplementary Fig. 10.** Photophysical properties of BTP **a,** Absorption and fluorescence spectra of the aqueous BTP solution. **b,** Cyclic voltammetry curves of BTP and blank at a scan rate of 10 mV∙s^−1^ in a three-electrode system. **c,** Ground and excited redox potential of BTP. The experiment was repeated three times independently, and each experiment showed similar results. Source data are provided as a Source Data file.


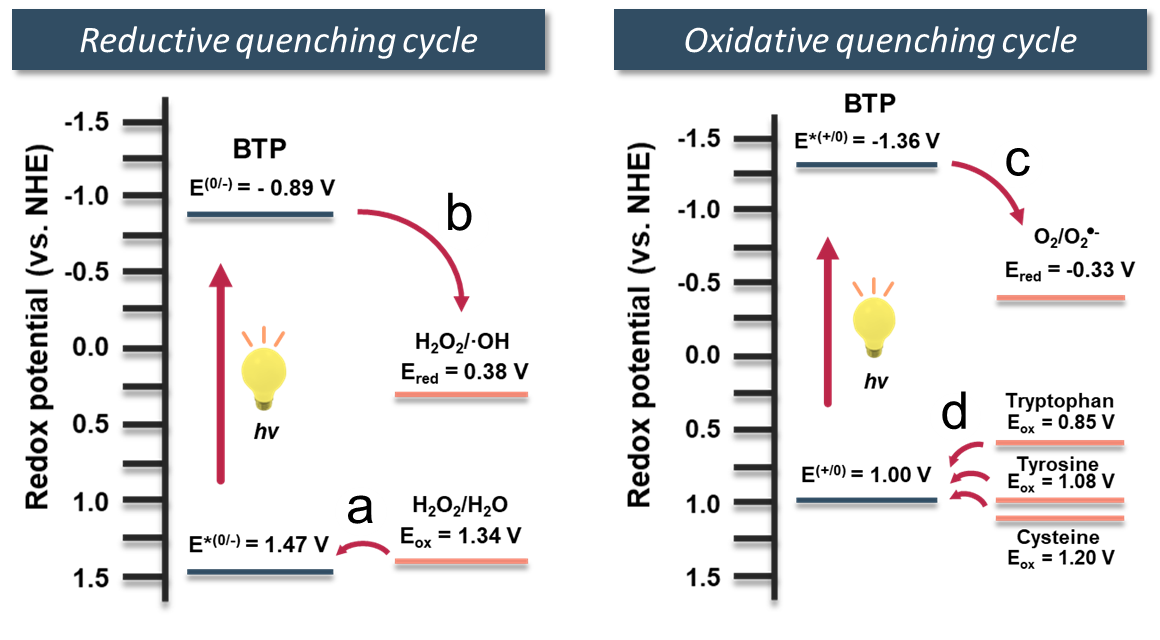


**Supplementary Fig. 11.** Proposed scheme of electron transfer for reductive and oxidative quenching cycles.


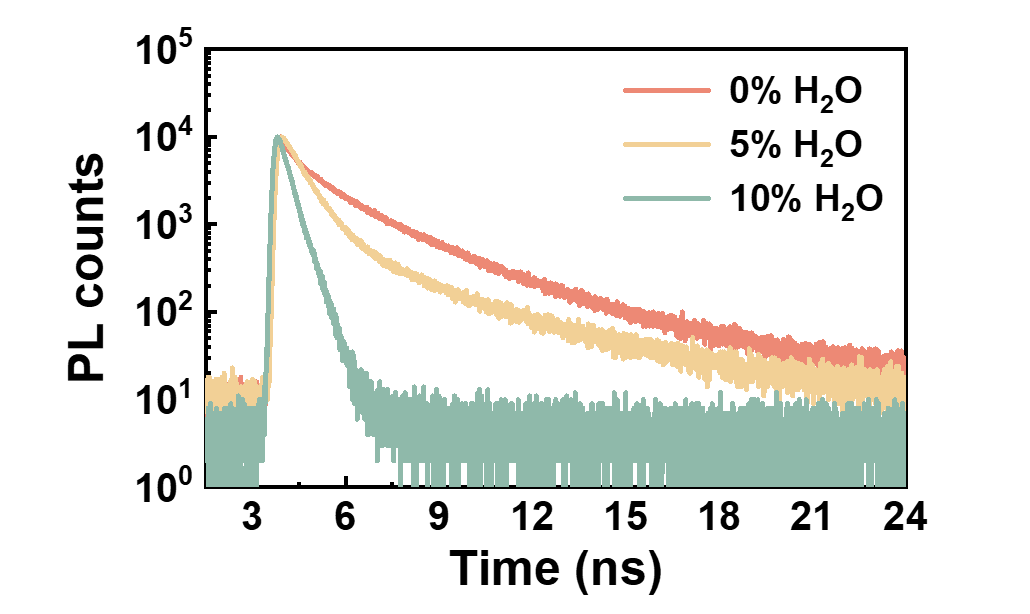


**Supplementary Fig. 12.** Time-correlated single-photon counting (TCSPC) spectra of BTP in acetonitrile containing various amounts of H_2_O (0, 5, and 10% H_2_O). The experiment was repeated three times independently, and each experiment showed similar results. Source data are provided as a Source Data file.


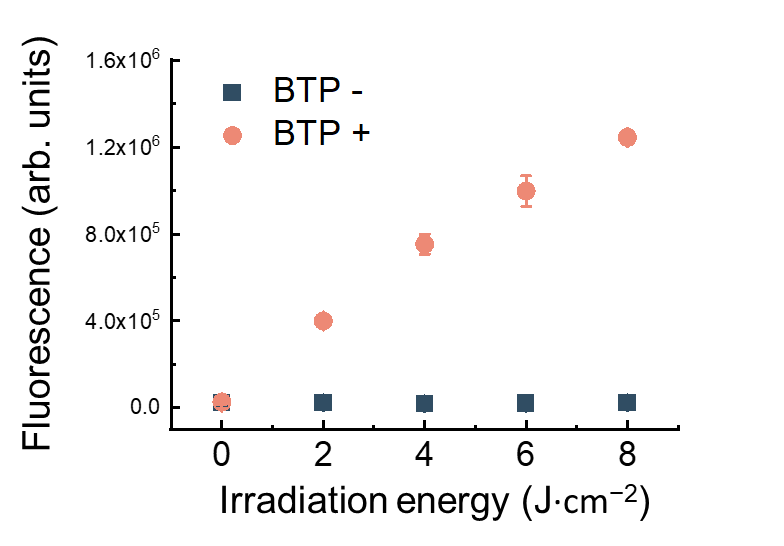


**Supplementary Fig. 13.** ∙OH generation assay using HPF. The results indicated a change in ∙OH generation with irradiation energy (blue LED, λ_max_ = 450 nm, 16.6 mW·cm^−2^). Data are presented as mean ± s.d. (*n* = 3 independent experiments). Source data are provided as a Source Data file.


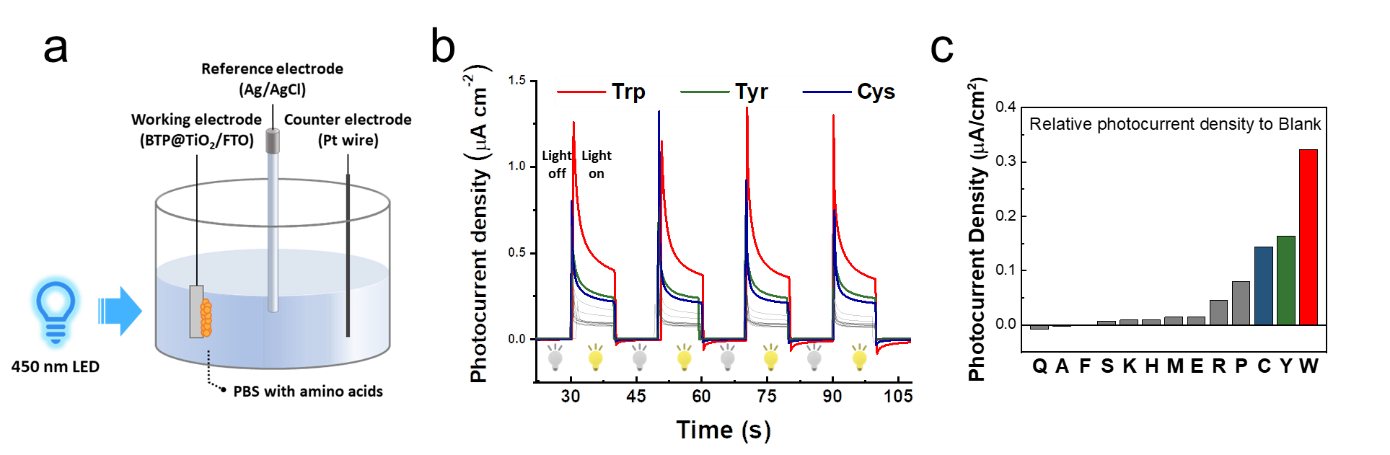


**Supplementary Fig. 14.** Electrochemical photooxidation of amino acids **a,** A three-electrode system for measuring the photocurrent by photocatalytic amino acid oxidation. **b,** Transient photocurrent responses depending on amino acids. Light exposure was turned on and off every 10 s and repeated. **c.** Relative photocurrent for each amino acid.


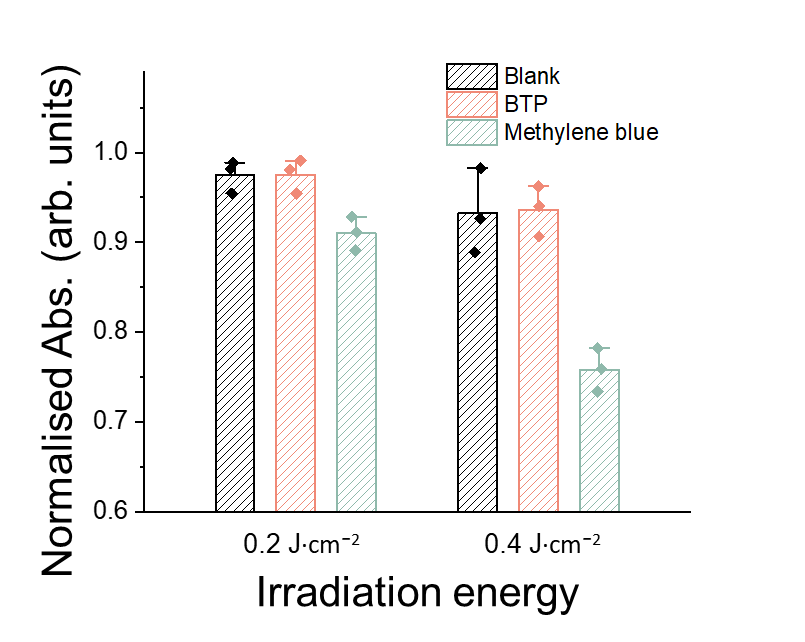


**Supplementary Fig. 15.** ^1^O_2_ generation assay using ABDA. The absorbance of ABDA (λ = 400 nm) was normalised to that observed under non-irradiated condition, and the decrease in the absorbance represents ^1^O_2_ generation. Methylene blue was used as a positive control. Data are presented as mean ± s.d. (*n* = 3 independent experiments). Source data are provided as a Source Data file.


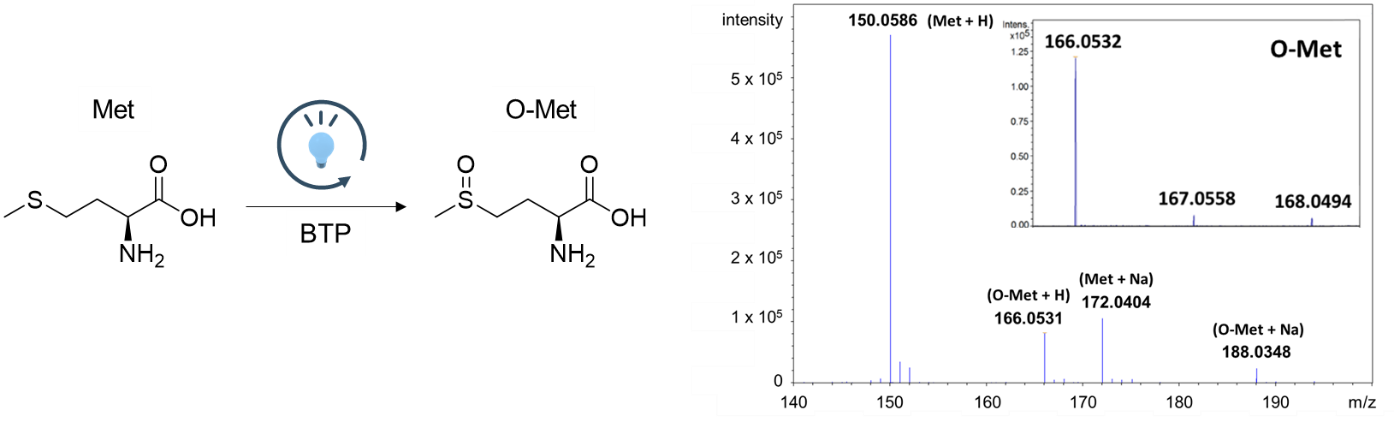


**Supplementary Fig. 16.** Methionine oxidation by BTP photocatalysis. Oxidised methionine (O-Met) was detected by HRMS. The inserted image exhibits isotopic distribution of [O-Met + H]. [BTP] = 100 μM, [Met] = 1 mM. A blue LED lamp was used to excite BTP (λ_max_ = 450 nm, 16.6 mW·cm^−2^ for 2 h).


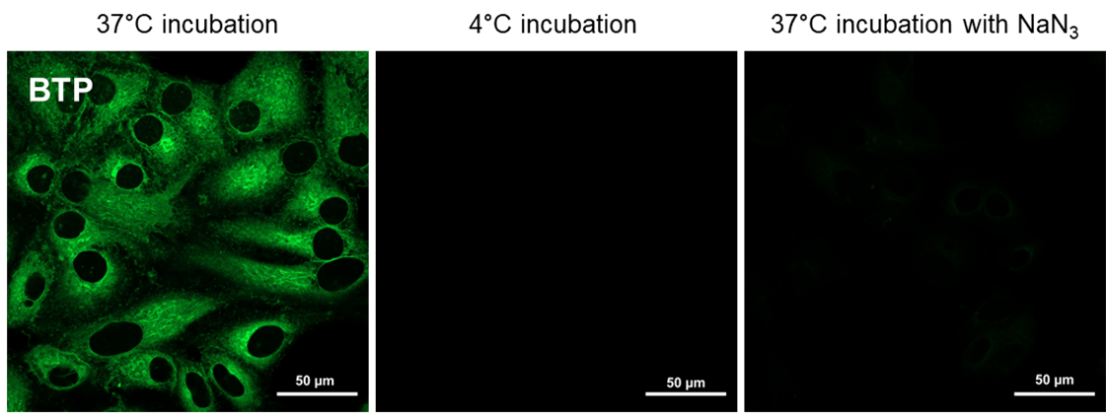


**Supplementary Fig. 17.** BTP uptake under physiological conditions at 37 ℃, at 4 ℃, and in the presence of NaN_3_. HeLa cells were pre-incubated at the conditions for 30 minutes, then further incubated with BTP (10 μM) for 2 hours. The experiment was repeated three times independently, and each experiment showed similar results.


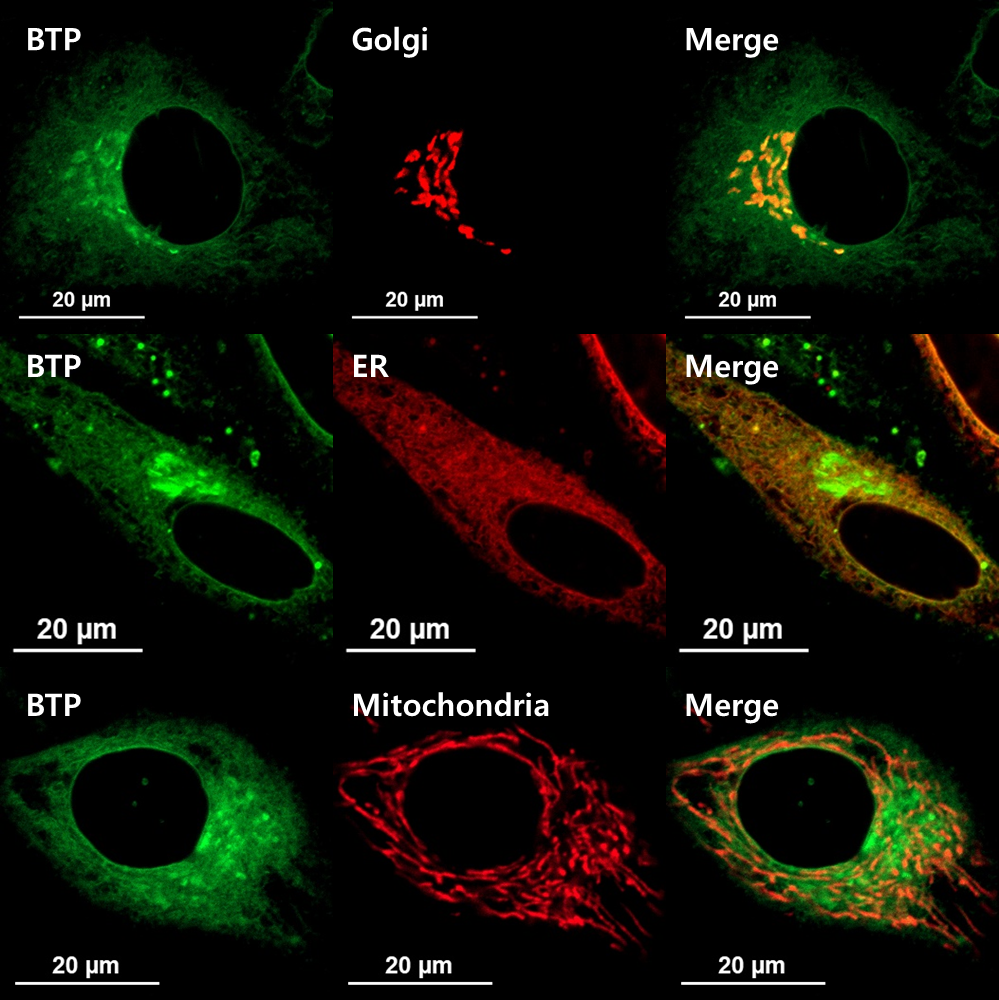


**Supplementary Fig. 18.** Co-localisation of BTP (5 μM). HeLa cells were transfected with CellLight™ Golgi RFP BacMam2.0 and Sec61b-mGFP constructs to stain the Golgi apparatus and endoplasmic reticulum (ER), respectively. MitoTracker^TM^ Deep Red FM was used to stain mitochondria. Fluorescence signals of GolgiRFP, Sec61b-mGFP, and MitoTracker are represented in red. The experiment was repeated three times independently, and each experiment showed similar results.


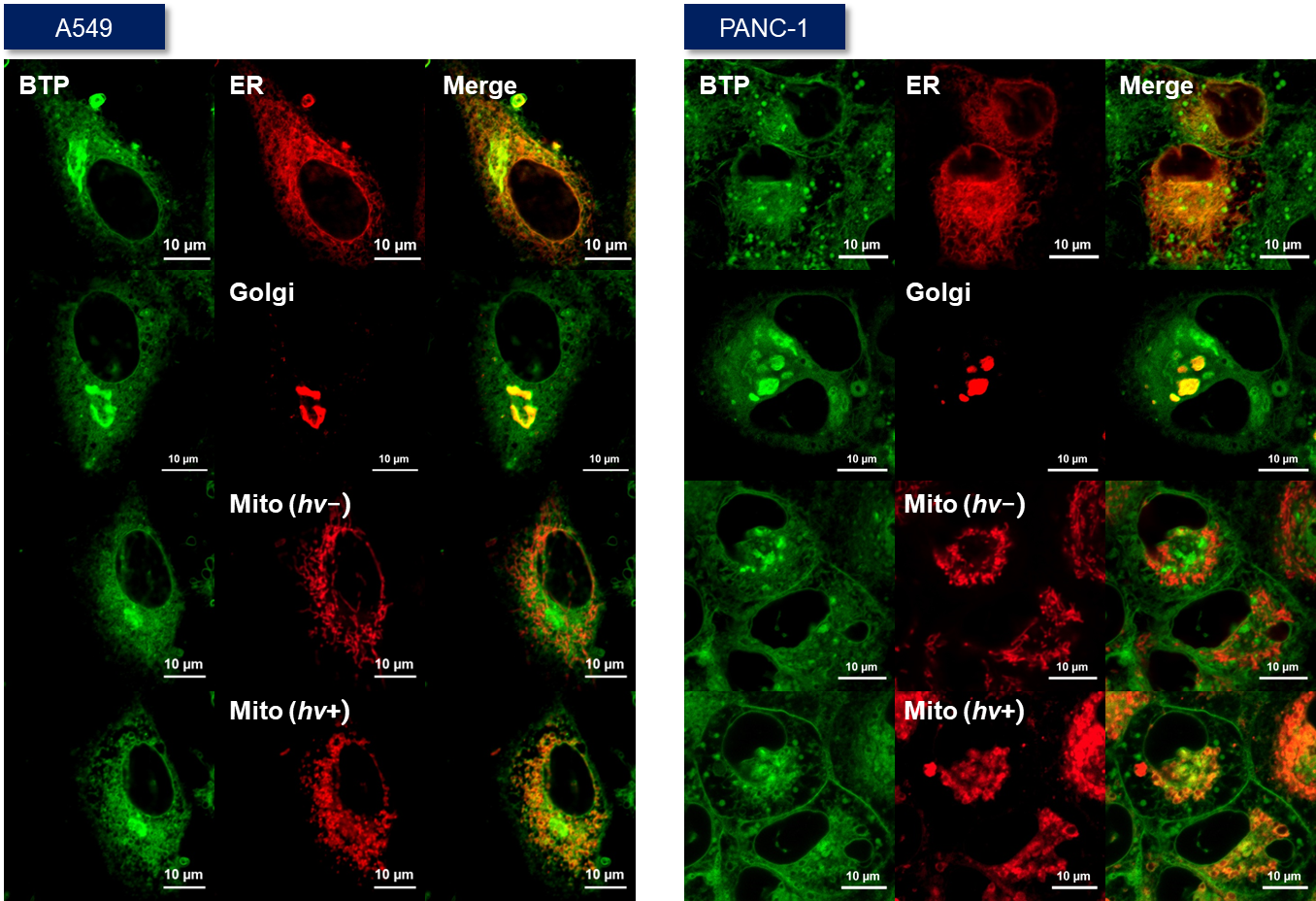


**Supplementary Fig. 19.** Co-localisation of BTP (5 μM). A549 and PANC-1 cells were transfected with CellLight™ Golgi RFP BacMam2.0 and Sec61b-mGFP constructs to stain the Golgi apparatus and endoplasmic reticulum (ER), respectively. Additionally, MitoTrackerTM Deep Red FM was used to stain mitochondria. The fluorescence signals of Golgi, ER, and MitoTracker are represented in red. Furthermore, re-localisation of BTP to mitochondria after photocatalysis was also observed. light exposure: confocal laser, λ = 445 nm. The experiment was repeated three times independently, and each experiment showed similar results.


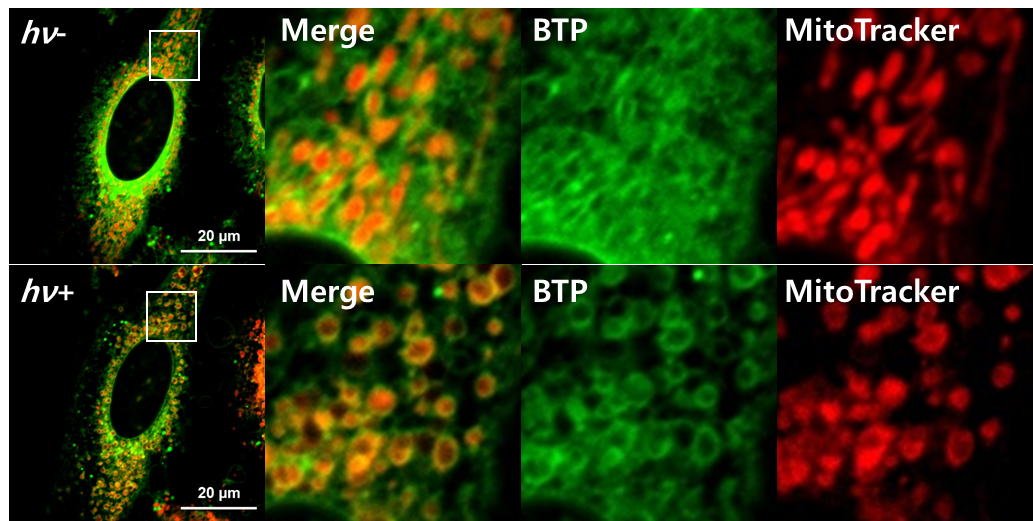


**Supplementary Fig. 20.** Changes in location of BTP after photocatalysis. HeLa cells were stained with BTP and MitoTracker and imaged before and after light exposure (confocal laser, λ = 445 nm). Enlarged images of white boxes show that BTP moves to the mitochondrial membrane during photocatalysis. The experiment was repeated three times independently, and each experiment showed similar results.


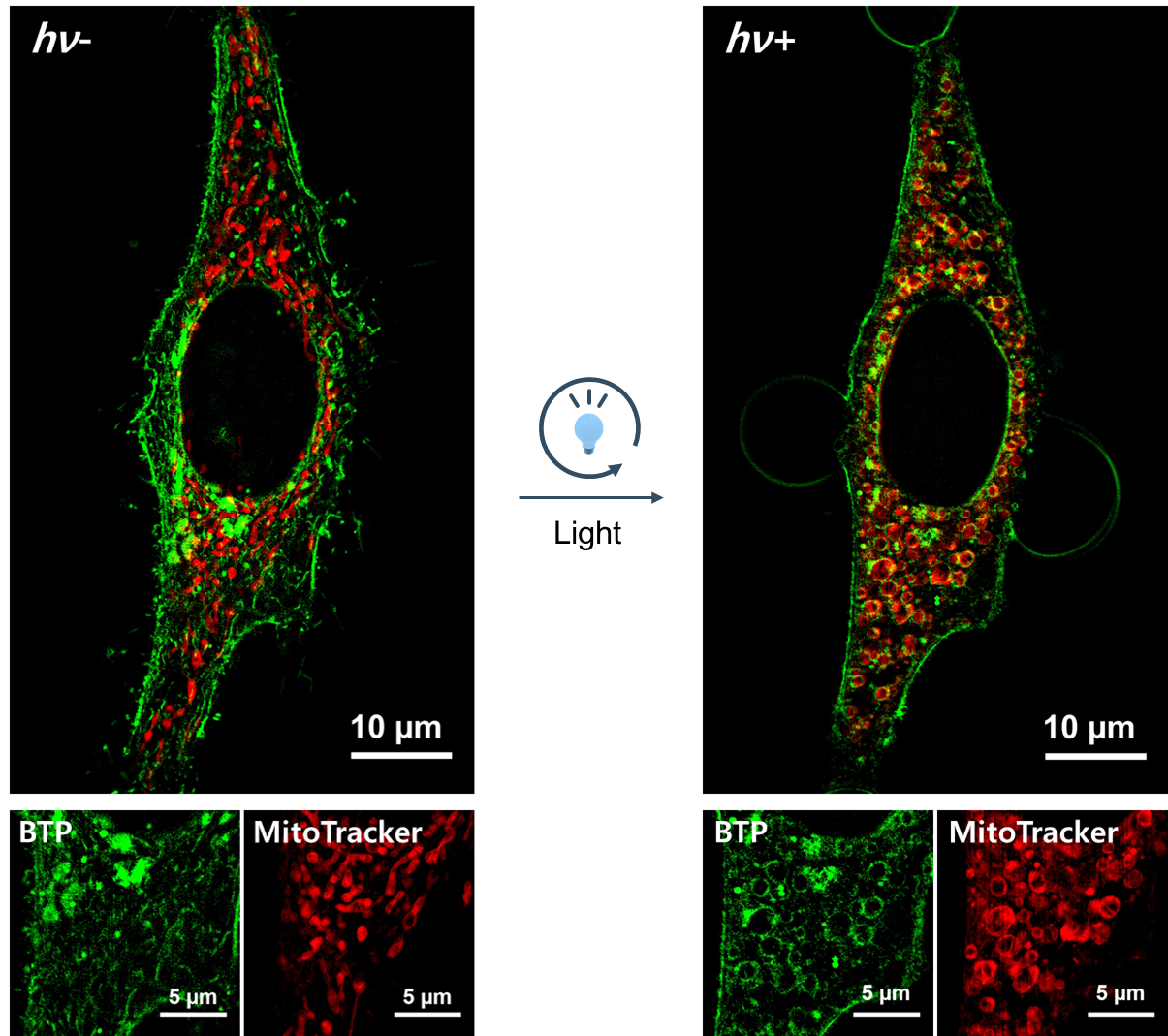


**Supplementary Fig. 21.** Live-structured illumination microscopy (live-SIM) images using HeLa cells before and after BTP photocatalysis (λ = 445 nm). The green and red signals correspond to BTP and MitoTracker, respectively. The experiment was repeated three times independently, and each experiment showed similar results.


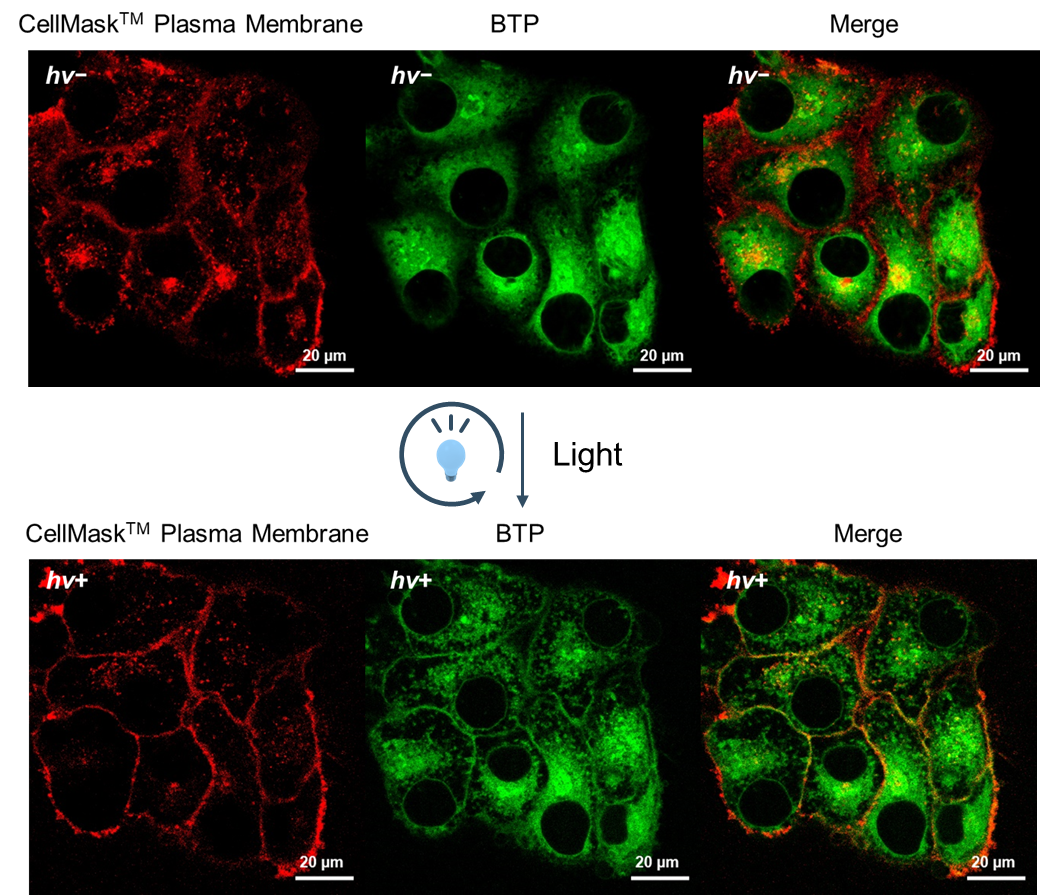


**Supplementary Fig. 22.** Co-localisation of BTP with a plasma membrane staining dye. HeLa cells were stained with BTP (10 μM for 2 hours) and CellMask^TM^ Deep Red Plasma Membrane Stain (5 μg∙mL^−1^ for 10 minutes). The HeLa cells were imaged before and after light exposure (confocal laser, λ = 445 nm). The experiment was repeated three times independently, and each experiment showed similar results.


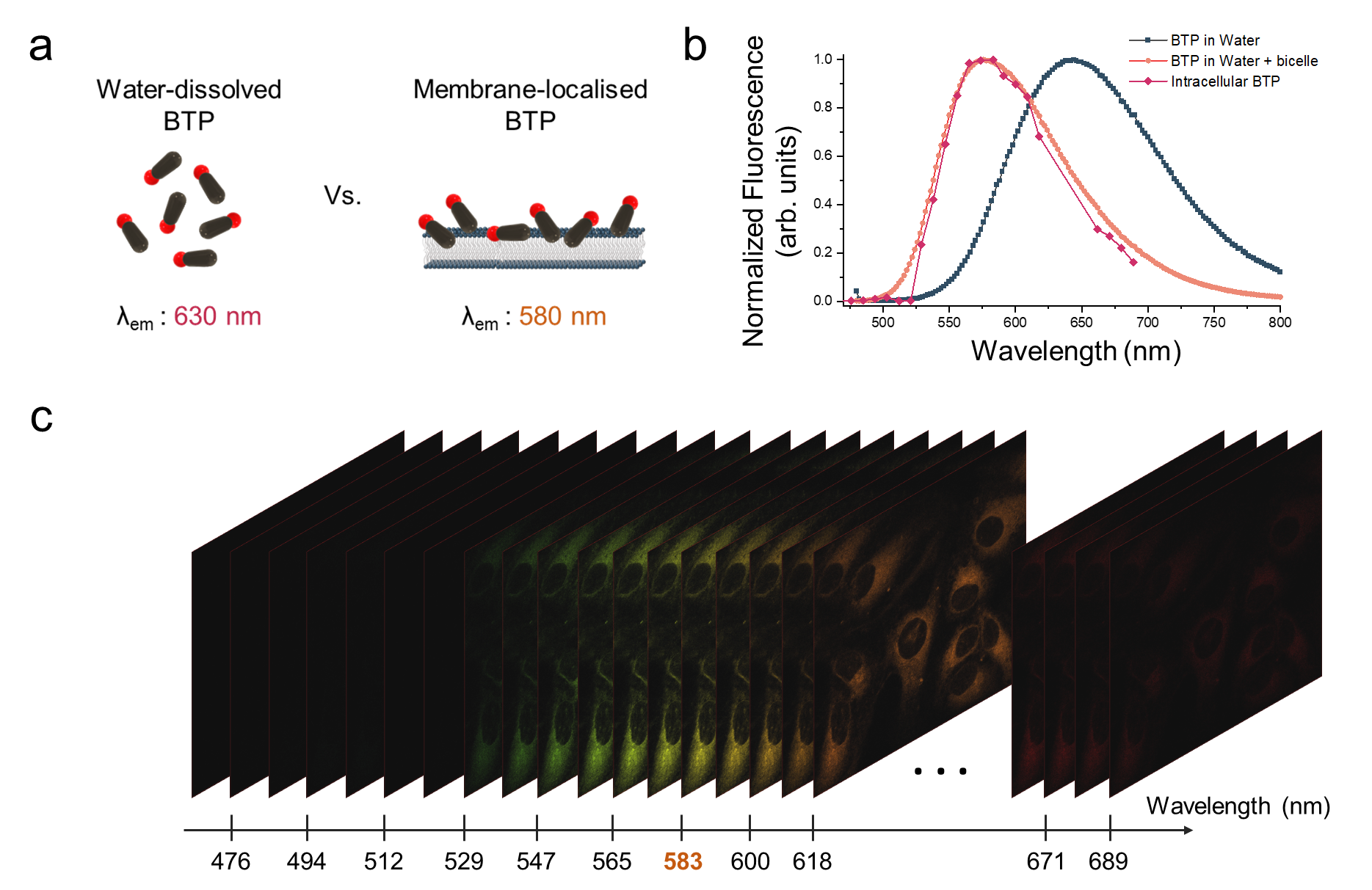


**Supplementary Fig. 23.** Intracellular membrane localisation of BTP and intracellular ROS generation. **a,** A schematic image of BTP fluorescence depending on surrounding environments. **b,** Normalised BTP fluorescence in different conditions. Blue: BTP in 50 mM Tris buffer (pH 7.5, [BTP] = 20 μM), orange: BTP in BTP in 50 mM Tris buffer (pH 7.5, [BTP] = 20 μM) + bicelles, red: intracellular environment (HeLa cells). The fluorescence of BTP inside cells were obtained by the Lambda-scan mode of confocal microscopy. **c,** Confocal images of BTP were taken at each wavelength, and merged was produced. The experiment was repeated three times independently, and each experiment showed similar results. Source data are provided as a Source Data file.


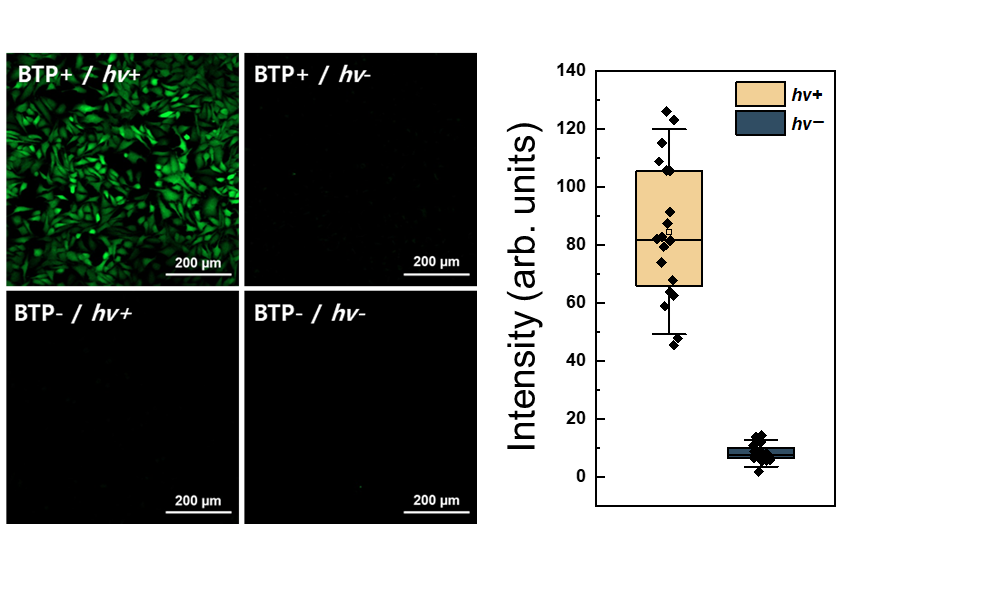


**Supplementary Fig. 24.** H_2_DCF-DA assay for intracellular ROS generation. HeLa cells were incubated with BTP (5 μM) and H_2_DCF-DA (20 μM) and irradiated with blue LED light (λ_max_ = 450 nm, 5 J·cm^−2^). The green signal corresponds to the DCF fluorescence. Box plot analysis of DCF signals from randomly selected 20 cells (BTP+/*hv*+ and BTP+/*hv*−). The whiskers represent the standard deviations (s.d.), and the box represents to 25% and 75% of the s.d. Data are presented as mean ± s.d. (*n* = 20 cells from three imaging plates). The experiment was repeated three times independently, and each experiment showed similar results. Source data are provided as a Source Data file.


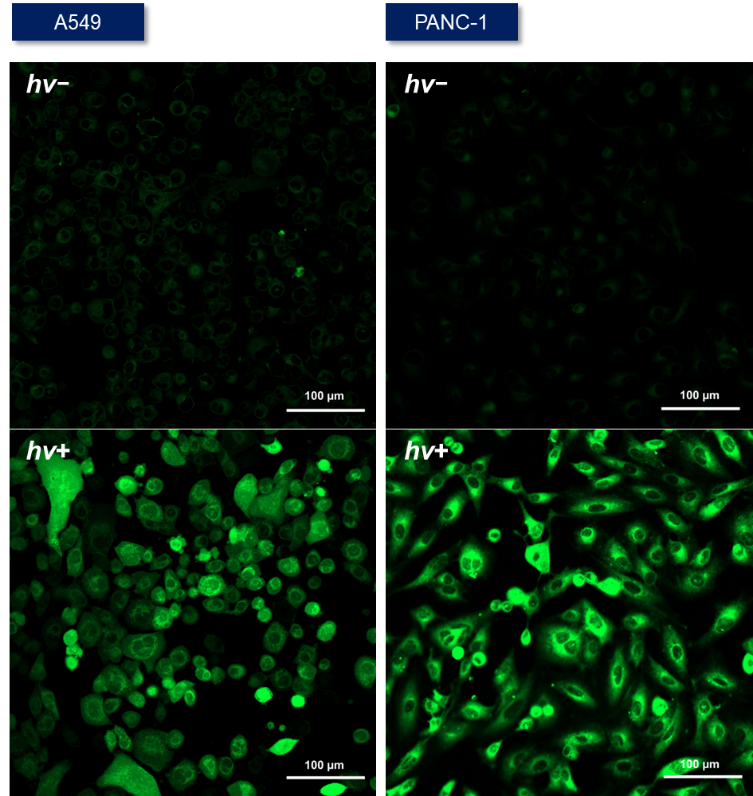


**Supplementary Fig. 25.** H_2_DCF-DA assay for intracellular ROS generation. A549 and PANC-1 cells were incubated with BTP (5 μM) and H_2_DCF-DA (20 μM), followed by irradiation with blue LED light (λ_max_ = 450 nm, 5 J∙cm^−2^). The green signal corresponds to DCF fluorescence. The experiment was repeated three times independently, and each experiment showed similar results.


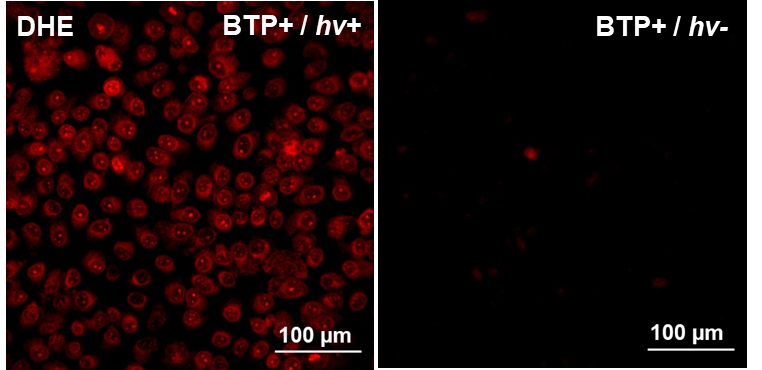


**Supplementary Fig. 26.** O_2_^−∙^ assay using dihydroethidium (DHE). HeLa cells were treated with DHE (5 μM) and exposed to BTP photocatalysis (λ_max_ = 450 nm, 10 J·cm^−2^). Red signals correspond to DNA-intercalated 2-hydroxyethidium. The experiment was repeated three times independently, and each experiment showed similar results.


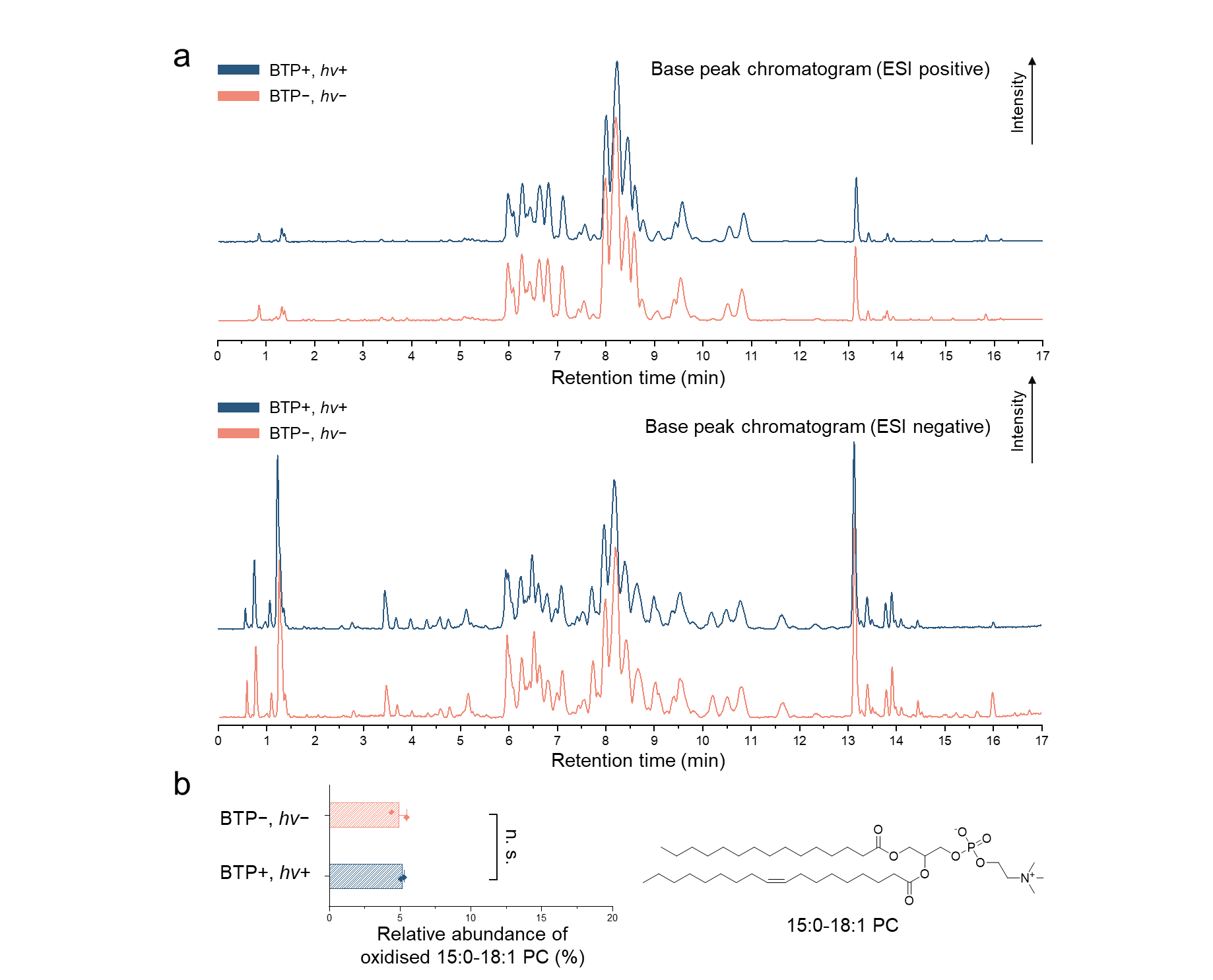


**Supplementary Fig. 27.** Lipid oxidation analysis using ultra-performance liquid chromatography-MS (UPLC-MS). **a,** Base peak chromatograms of lipid extracts from HeLa cells (BTP+/*hv*+ and BTP−/*hv*−) for ESI positive and negative mods. The HeLa cells were incubated with BTP (5 μM) and irradiated with blue LED light (λ_max_ = 450 nm, 10 J·cm^−2^), then total lipids of the cells were extracted using Folch’s method. The lipid extracts were analysed by UPLC-MS. To identify 15:0-18:1 phosphatidylcholine (PC), SPLASH LipidoMIX^TM^ Internal Standard was added to the lipid extracts. **b,** The relative abundance of oxidised 15:0-18:1 PC and the molecular structure of 15:0-18:1 PC. Relative abundances are calculated as A_O-PC_/(A_O-PC_+A_PC_), where A_O-PC_ represents the retention time area of oxidised 15:0-18:1 PC and A_PC_ represents the retention time area of native 15:0-18:1 PC. Data are presented as mean ± s.d. *P* = 0.7 (*n* = 2 biologically independent samples). Source data are provided as a Source Data file.

**
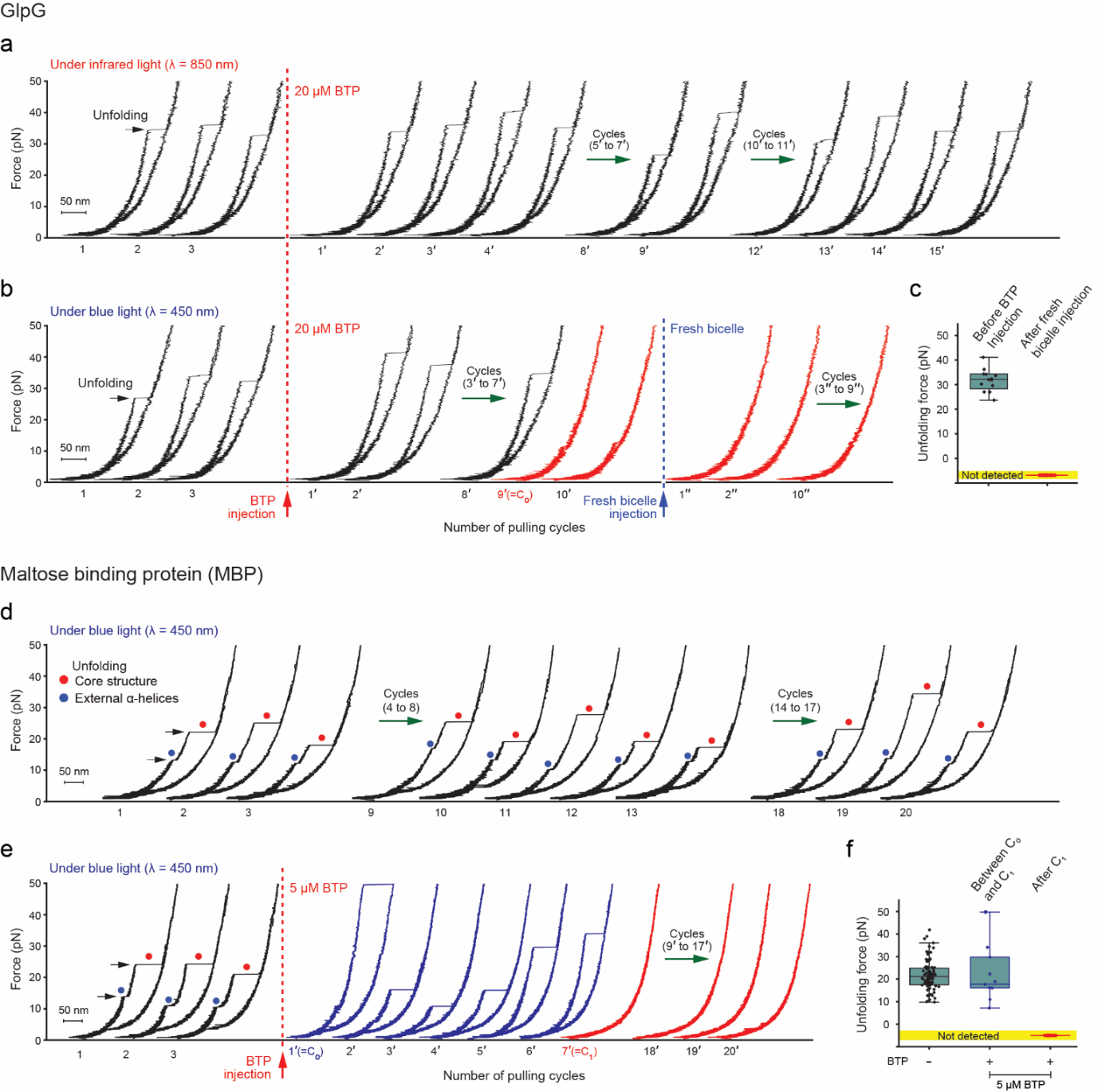
**

**Supplementary Fig. 28.** Single-molecule forced-unfolding assay for control conditions. **a,** Representative force-extension curves of GlpG in the condition of infrared light exposure (λ_peak_ = 850 nm, 39.51 mW·cm^−2^). Under the infrared light, the C_0_ point of the first abnormal unfolding was not observed even after tens of cycles upon BTP addition (for blue light condition as shown in Fig. 2h, average C_0_ = 4′ cycles). This result is because the BTP is not able to absorb the energy of infrared wavelength. **b,** Representative force-extension curves of GlpG in the condition of blue light exposure and fresh bicelle injection. After the C_0_ point, the solution of fresh bicelles is injected by buffer exchange. **c,** Unfolding forces observed in the conditions before the injection of BTP (*n* = 12 independent samples) and after the injection of fresh bicelles (*n* = 30 independent samples). The results of panels b and c show that the normal unfolding is not recovered even after the exchange of fresh bicelles. This indicates that the drastic reduction of unfolding forces is attributed to the oxidative damage on the structure/fold of the membrane protein, at least. This type of experiments does not confirm a damaging effect on the bicelles, although it is possible. Data are presented as mean ± s.d. Source data are provided as a Source Data file.


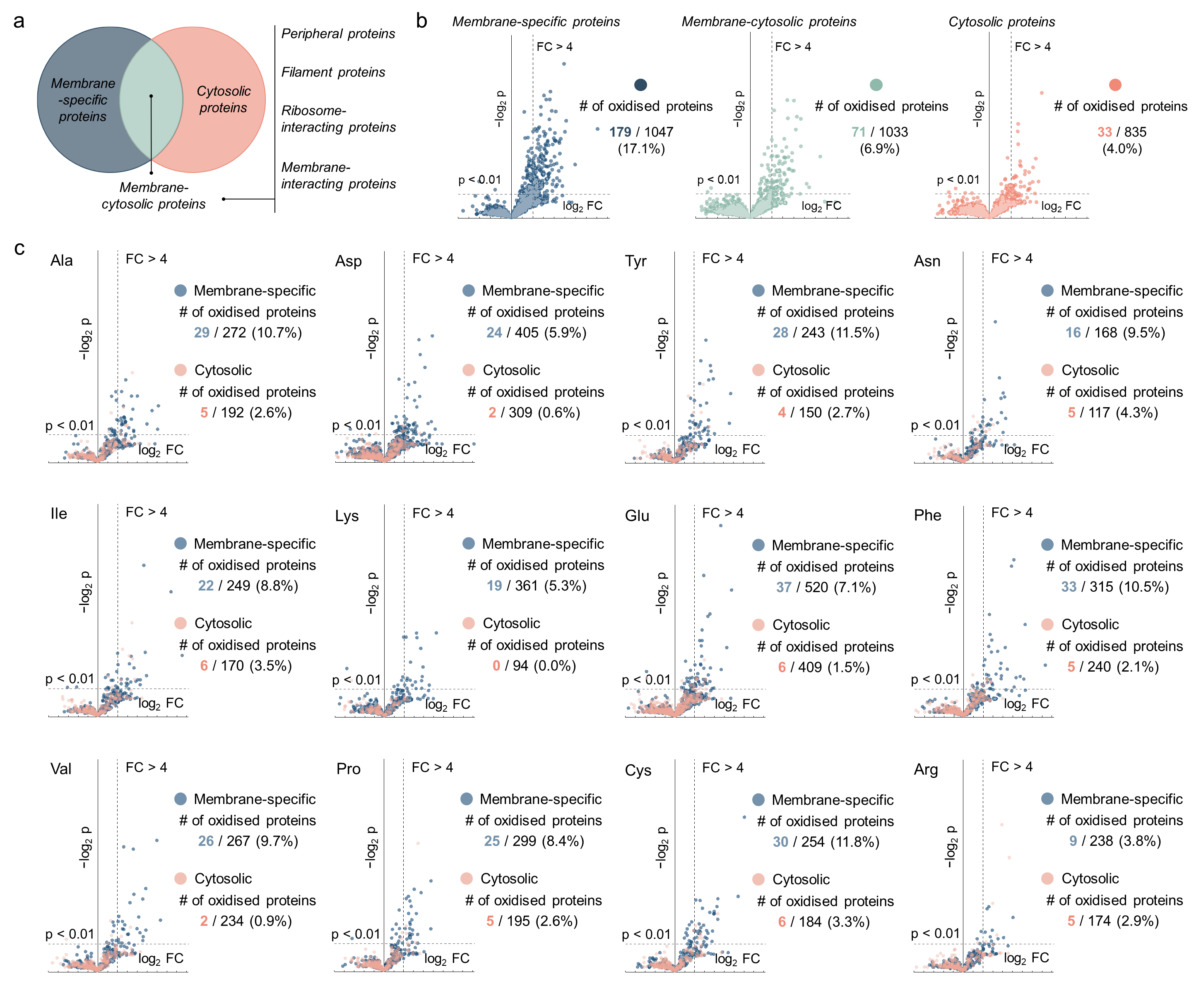


**Supplementary Fig. 29.** Volcano plots for oxidative modifications of each amino acid. **a,** Classification of proteins based on Gene Ontology (GO) subcellular annotations: 'Membrane-specific' proteins are exclusively located on membranes, while 'membrane-cytosol' proteins are present on both membranes and in the cytosol. Proteins localized in the cytosol are referred to as 'cytosolic' proteins. **b,** Volcano plots depicting the classification of proteins based on detected MS spectra intensities of oxidative modifications for 17 amino acids. **c,** Comparison of protein oxidation between membrane-specific and cytosolic proteins, focusing on amino acids not highlighted in the main figures. All P values were calculated for student’s one-tailed t-test. Source data are provided as a Source Data file.


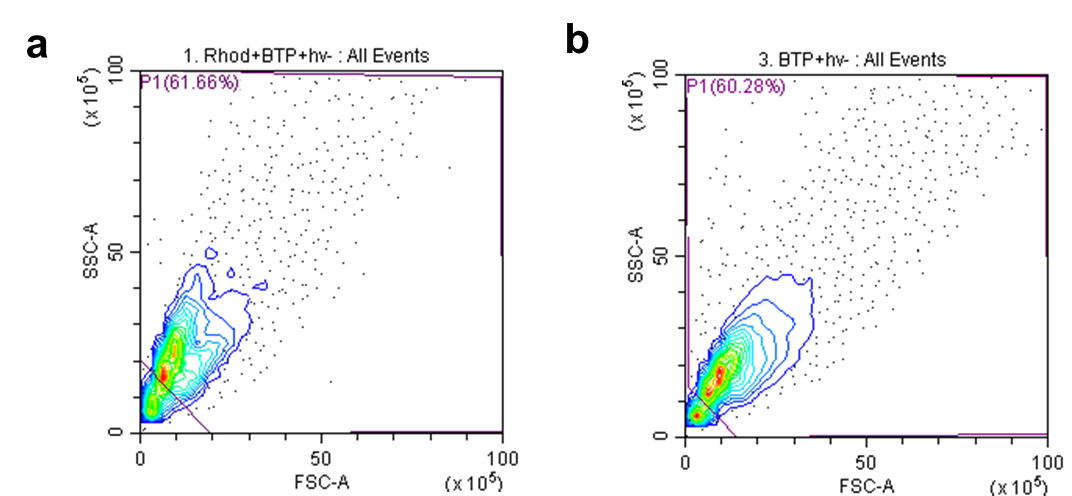


**Supplementary Fig. 30.** Gating strategies for flow cytometry experiments. Cell size (forward scattering area, FSC-A) and granularity (side scatter area, SSC-A) were used to filter the cell debris for **a.** Rhod-2 assay (for Fig. 5d) **b.** and Ion K+ green-2 assay (for Fig. 5g). We discarded cells with extremely low signal of FSC and SSC. The boundaries of FSC and SSC were 2 × 10^6^ and 1.5 × 10^6^, respectively (Areas separated by purple lines).


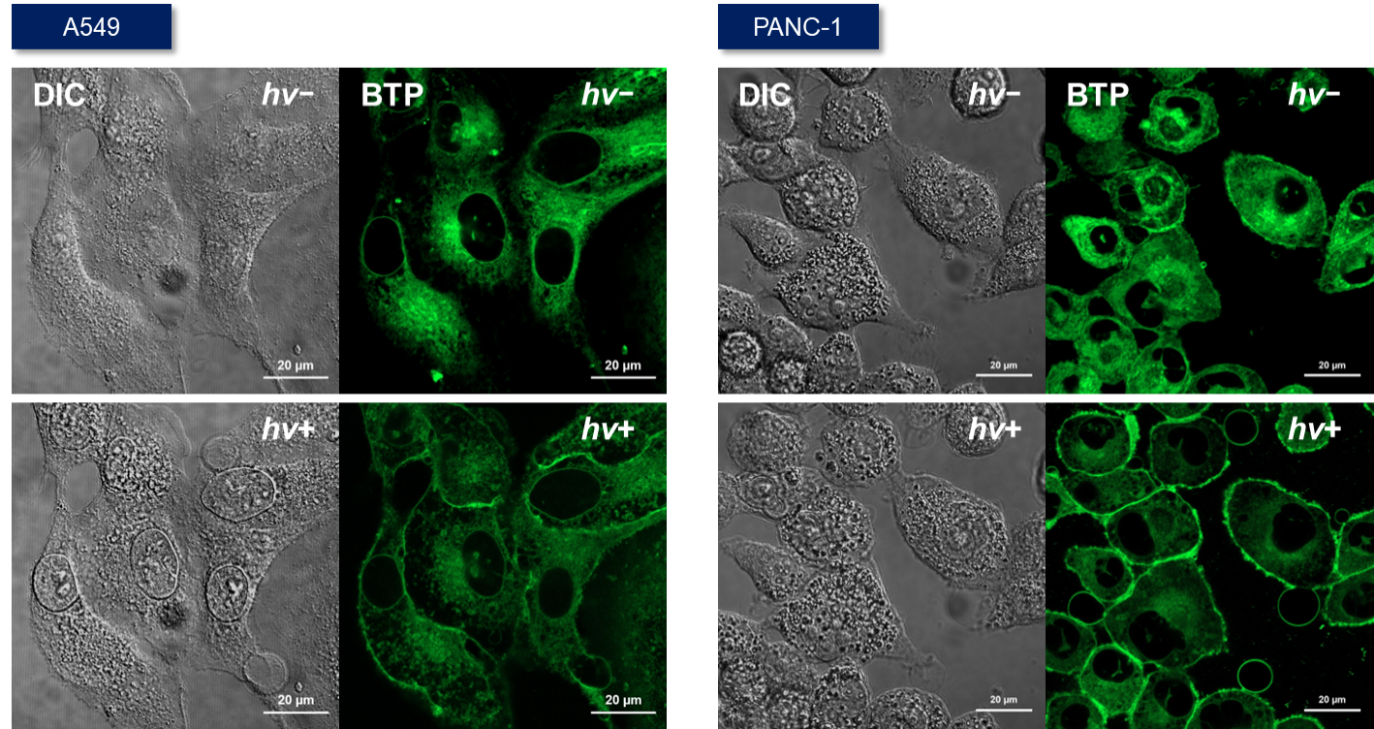


**Supplementary Fig. 31.** Pyroptotic morphology changes of A549 and PANC-1 cells in response to photocatalytic membrane oxidation. The cells were incubated with BTP (10 μM) for 2 hours. light exposure: confocal laser, λ = 445 nm. The experiment was repeated three times independently, and each experiment showed similar results.


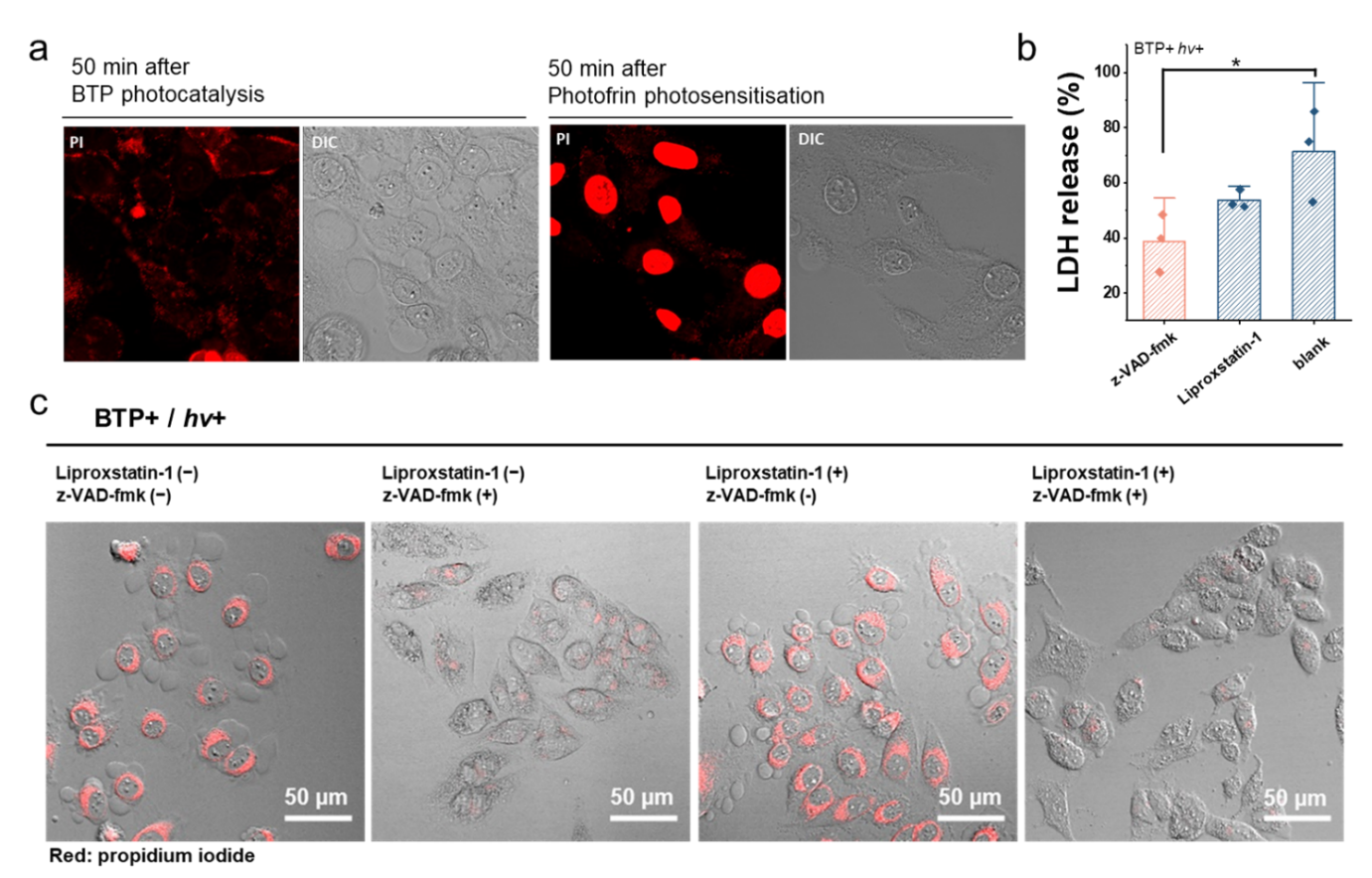


**Supplementary Fig. 32.** Cell death modality induced by BTP photocatalysis. **a,** PI staining after BTP photocatalysis. The PI fluorescence of HeLa cells was not observed in the nucleus 50 min after BTP photocatalysis, implying that the nuclear envelope was intact. During pyroptosis, in which the nuclear membrane is retained, PI could not penetrate the nuclear envelope (top). Photofrin (an apoptosis and necroptosis inducer) was used as the control photosensitiser (bottom). The experiment was repeated three times independently, and each experiment showed similar results. **b,** LDH assay of HeLa cells with/without liproxstatin-1 (ferroptosis inhibitor, 10 µM) or z-VAD-fmk (pan-caspase inhibitor, 4 µM) 2 hours after BTP photocatalysis (λ_max_ = 450 nm, 3 J·cm^−2^) (*n* = 3 independent samples). **c,** Morphological changes and propidium iodide (PI) staining of HeLa cells with and without liproxstatin-1 or z-VAD-fmk after BTP photocatalysis (λ_max_ = 450 nm, 3 J·cm^−2^). BTP photocatalysis induced pyroptotic blebbing of cells without z-VAD-fmk. The experiment was repeated three times independently, and each experiment showed similar results. Note that PI fluorescence was enhanced in cells with pyroptotic blebbing owing to plasma membrane permeabilisation. All data are presented as mean ± s.d. (*n* = 3 independent samples) **P* = 0.046. Student’s two-tailed *t*-test. Source data are provided as a Source Data file.


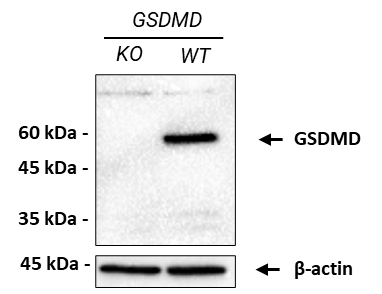


**Supplementary Fig. 33.** GSDMD expression level of GSDMD knocked-out (left) or wild-type (right) iBMDM was assessed by immunoblot. The experiment was repeated three times independently, and each experiment showed similar results. Source data are provided as a Source Data file.


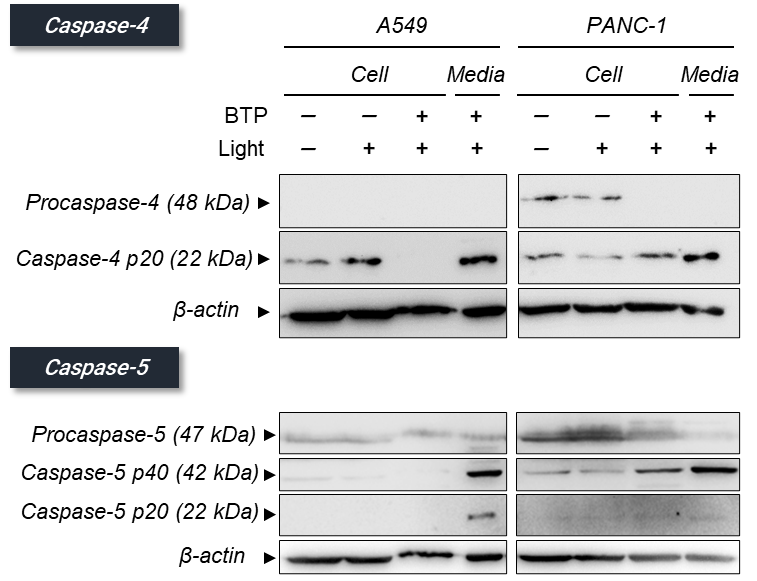


**Supplementary Fig. 34.** Western blot analysis of A549 and PANC-1 cells with BTP photocatalysis for investigating caspase-4/5 cleavage. The experiment was repeated three times independently, and each experiment showed similar results. Source data are provided as a Source Data file.


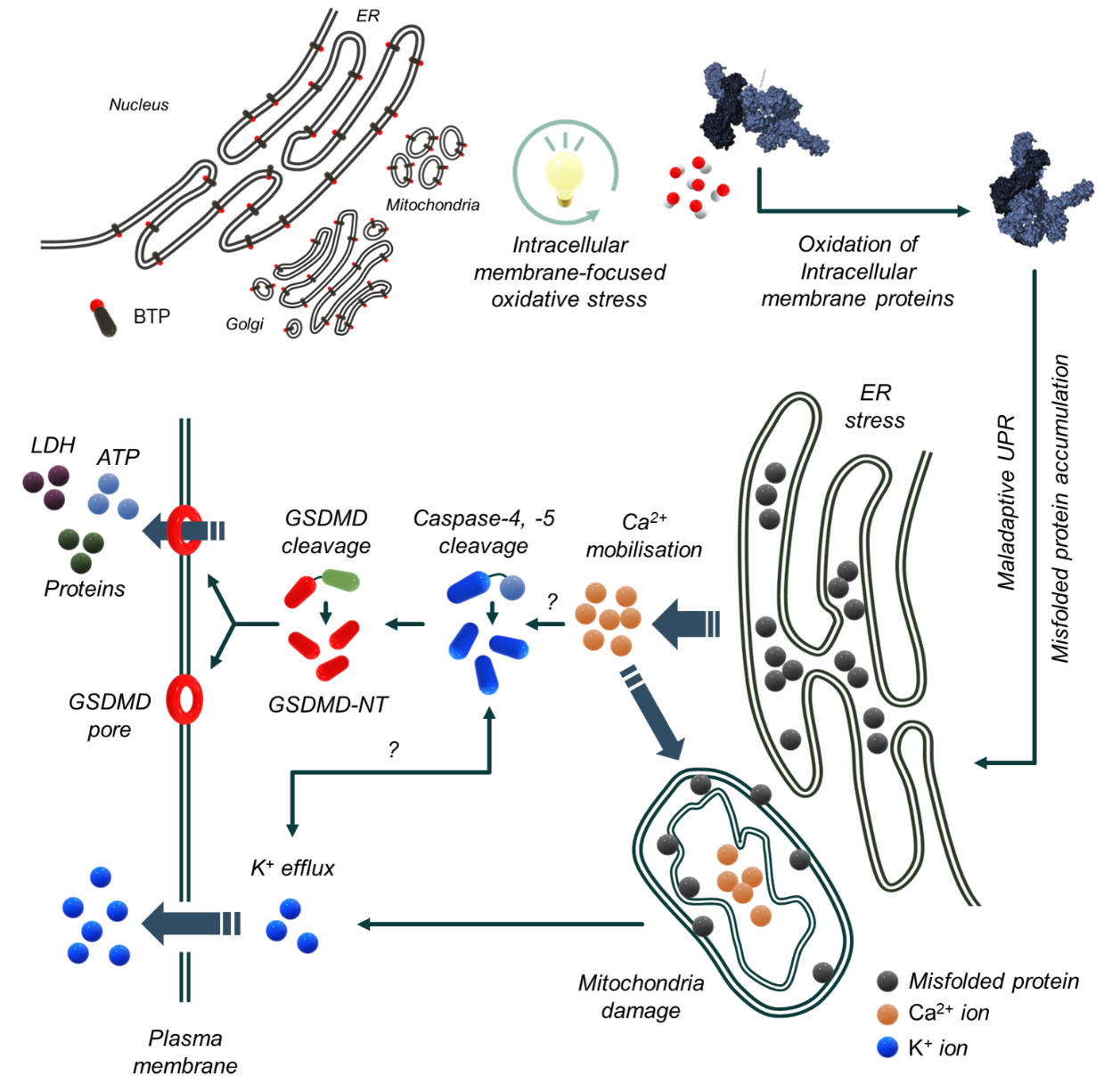


**Supplementary Fig. 35.** Proposed mechanism of non-canonical pyroptosis induced by photocatalytic membrane oxidation. The oxidative photocatalysis of BTP generates hydroxyl radicals, hydrogen peroxide, and superoxide radicals on intracellular membranes. Severe oxidative stress on the membrane causes misfolding of membrane proteins in the ER, Golgi apparatus, and mitochondria. Notably, the oxidation of protein quality control (PQC)-related proteins induces ER stress and maladaptive UPR attributed to the accumulation of misfolded proteins. During the maladaptive UPR process, Ca^2+^ is released from the ER lumen. Simultaneously, mitochondrial damage caused by membrane oxidation results in K^+^ efflux. Cation mobilisation and ER stress presumably cleave caspase-4/5, leading to GSDMD pore formation. Through the GSDMD pore, biomolecules are released from the cytosol, triggering non-canonical pyroptosis.

**Supplementary Table 1.** Modifications considered during the second search to identify oxidized amino acids. **a.** MS-common modifications provided by MODplus **b.** Modifications caused by cell FPOP. The overlapping ones between **a** and **b** were only considered once (as FPOP-related modifications) during the search.


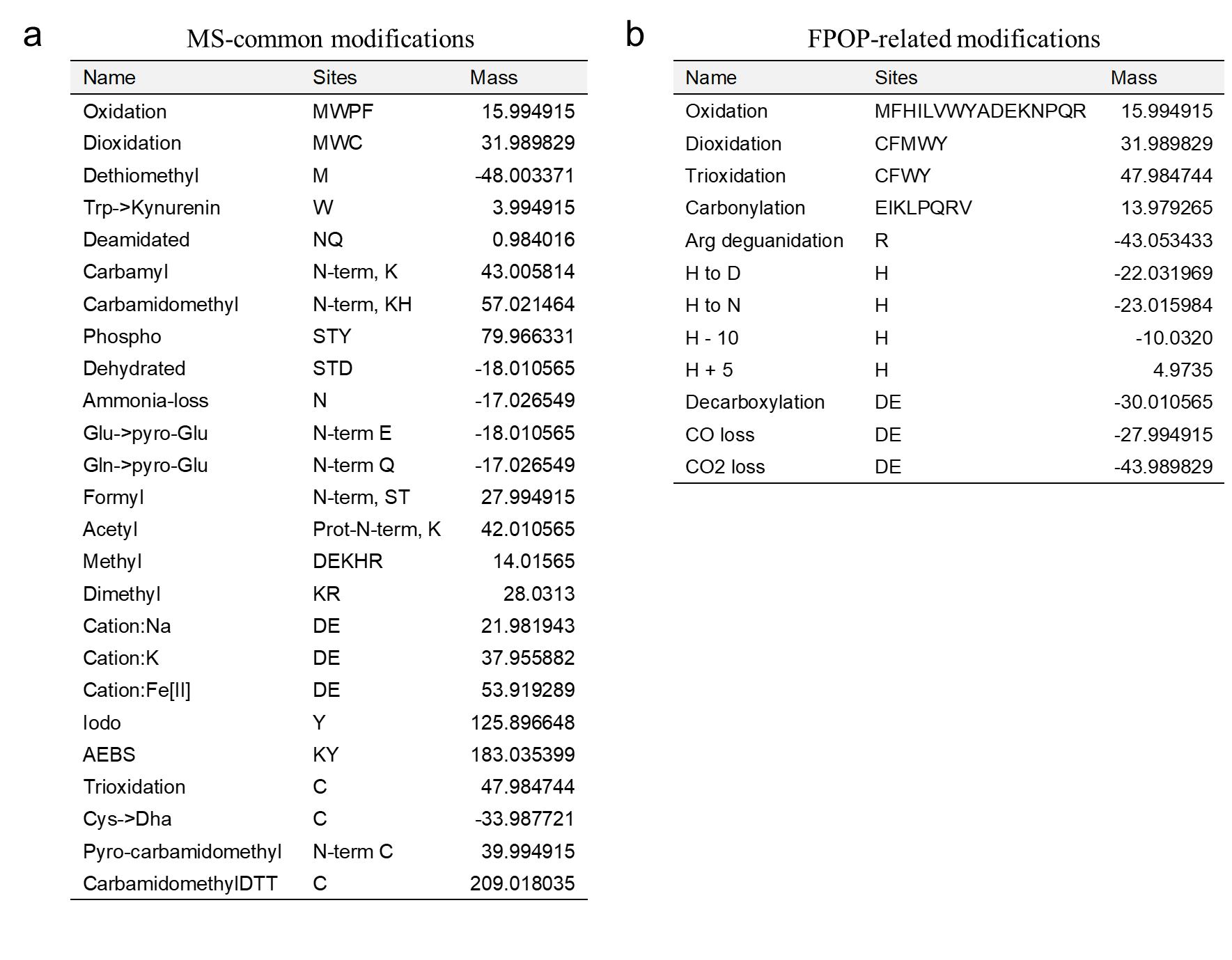


**Supplementary References**

1 Roh, D. H. *et al.* Strategy for Improved Photoconversion Efficiency in Thin Photoelectrode Films by Controlling pi-Spacer Dihedral Angle. *J Phys Chem C* **120**, 24655-24666 (2016).
