# Supplementary material for: Oxidative photocatalysis on membranes triggers non-canonical pyroptosis
Source: Nat Commun. 2024 May 13;15:4025. doi: 10.1038/s41467-024-47634-5 (PMC11091103; doi:10.1038/s41467-024-47634-5)
Supplement: Supplementary file 2 — Reporting Summary [file 41467_2024_47634_MOESM2_ESM.pdf]

nature portfolio

Corresponding author(s): Tae-Hyuk Kwon

Last updated by author(s): Mar 28, 2024

Reporting Summary

Nature Portfolio wishes to improve the reproducibility of the work that we publish. This form provides structure for consistency and transparency in reporting. For further information on Nature Portfolio policies, see our Editorial Policies and the Editorial Policy Checklist.

Statistics

For all statistical analyses, confirm that the following items are present in the figure legend, table legend, main text, or Methods section.

n/a	Confirmed	
☐	☒	The exact sample size (*n*) for each experimental group/condition, given as a discrete number and unit of measurement
☐	☒	A statement on whether measurements were taken from distinct samples or whether the same sample was measured repeatedly
☐	☒	The statistical test(s) used AND whether they are one- or two-sided<br>*Only common tests should be described solely by name; describe more complex techniques in the Methods section.*
☒	☐	A description of all covariates tested
☒	☐	A description of any assumptions or corrections, such as tests of normality and adjustment for multiple comparisons
☐	☒	A full description of the statistical parameters including central tendency (e.g. means) or other basic estimates (e.g. regression coefficient) AND variation (e.g. standard deviation) or associated estimates of uncertainty (e.g. confidence intervals)
☐	☒	For null hypothesis testing, the test statistic (e.g. *F*, *t*, *r*) with confidence intervals, effect sizes, degrees of freedom and *P* value noted<br>*Give P values as exact values whenever suitable.*
☒	☐	For Bayesian analysis, information on the choice of priors and Markov chain Monte Carlo settings
☒	☐	For hierarchical and complex designs, identification of the appropriate level for tests and full reporting of outcomes
☒	☐	Estimates of effect sizes (e.g. Cohen's *d*, Pearson's *r*), indicating how they were calculated

Our web collection on statistics for biologists contains articles on many of the points above.

Software and code

Policy information about availability of computer code

Data collection	ZEN software (ver. 3.0) from Carl Zeiss was utilized for collection of confocal and live SIM imaging.<br>LC-MS/MS data acquisition software Xcalibur (ver. 4.3) from Thermofisher Scientific was utilized.<br>To investigate FPOP modification, modification search tool MODplus (ver. 1.02) was used (Robust and Unrestrictive Identification of Post-Translational Modifications Using Mass Spectrometry. Anal. Chem. 91, 11324-11333 (2019)).<br>All MS identifications were subsequently rescored by Percolator (v3.06)(Semi-supervised learning for peptide identification from shotgun proteomics datasets. Nat. Methods. 4, 923-925 (2007)) and validated at an estimated FDR of 1%
Data analysis	For microscopy Imaging, Carl Zeiss ZEN 3.0 blue edition was used for analyzing and editing images.<br>All MS/MS samples were analysed using Sequest Sorcerer platform (Sagen-N Research, San Jose, CA, USA). Sequest was set up to search the Homo sapiens (20612 entries, UniProt (http://www.uniprot.org)). Scaffold Q+ (version 5.1.0, Proteome Software Inc., Portland, OR) was used to validate MS/MS based peptide and protein identifications. A peptide of greater than 99% probability for achieving FDR lower than 1.0% based no Scaffold Local FDR algorithm was accepted as a true identification. A protein identification of greater than 14.0% probability to achieve an FDR less than 1.0% and containing 2 or more identified peptides was accepted. Protein Prophet algorithm was used to calculate the protein probabilities. The GO annotations for the proteins were retrieved from NCBI (downloaded Feb 11, 2021).<br>Normalization was performed iteratively on intensities while medians being used for averaging. Of 5173389 spectra in the experiment at the given thresholds, 2120870 (41%) were included in quantification. A new distribution was created by Gaussian distribution with a downshift of 1.8 and width of 0.3 standard deviations. All processes were conducted using Perseus software platform from Max Planck Institute of Biochemistry. The pre-determined significantly oxidized proteins based on their log2FC and log2P were analyzed by GOnet, and oxidized proteome networks by functional association were determined by STRING and visualized by Gephi (ver. 0.9.2) or Cytoscape (ver. 3.9.1).

To comprehensively identify peptides including all oxidized amino acids, we employed a modification search tool, MODplus (v1.02). All identifications were subsequently rescored by Percolator (v3.06).

Further detailed methods are described in Methods section of manuscript.
For editing raw data, OriginPro 2017, Microsoft office 2017 were used.
The FACS data was processed by the CytExpert software (ver. 2.4.0.28)

For manuscripts utilizing custom algorithms or software that are central to the research but not yet described in published literature, software must be made available to editors and reviewers. We strongly encourage code deposition in a community repository (e.g. GitHub). See the Nature Portfolio guidelines for submitting code & software for further information.

Data

Policy information about availability of data

All manuscripts must include a data availability statement. This statement should provide the following information, where applicable:

- Accession codes, unique identifiers, or web links for publicly available datasets
- A description of any restrictions on data availability
- For clinical datasets or third party data, please ensure that the statement adheres to our policy

The authors declare that the data supporting the findings of this study are available within the article and its Supplementary Information. The protein information utilized in this study is available from the Homo sapiens protein sequence database (20612 entries, UniProt (http://www.uniprot.org)). Raw mass spectrometry dataset used for oxidised proteome analysis have been deposited to the ProteomeXchange Consortium via the PRIDE partner repository under accession code PXD038746 and project DOI 10.6019/PXD038746. All other data are provided in the main text, supplementary information, or from the corresponding author upon request. Source data are provided with this paper.

Research involving human participants, their data, or biological material

Policy information about studies with human participants or human data. See also policy information about sex, gender (identity/presentation), and sexual orientation and race, ethnicity and racism.

Reporting on sex and gender	N/A
Reporting on race, ethnicity, or other socially relevant groupings	N/A
Population characteristics	N/A
Recruitment	N/A
Ethics oversight	N/A

Note that full information on the approval of the study protocol must also be provided in the manuscript.

Field-specific reporting

Please select the one below that is the best fit for your research. If you are not sure, read the appropriate sections before making your selection.

[x] Life sciences [ ] Behavioural & social sciences [ ] Ecological, evolutionary & environmental sciences

For a reference copy of the document with all sections, see nature.com/documents/nr-reporting-summary-flat.pdf

Life sciences study design

All studies must disclose on these points even when the disclosure is negative.

Sample size	The calculation for sample size was not performed. For all experiments, sample sizes were determined based on similar experiments described in published articles (Nat. Commun. 12. 26 (2021)).
Data exclusions	No data were excluded from the analyses
Replication	All reported results were triplicated independently. Each attempts showed similar results.
Randomization	For the experiments using cell lines, we randomly allocated cells cultured in the same condition into experimental sets, including positive/negative control. In imaging analysis, we randomly selected target at least three individual cells, then we choosed the best images.
Blinding	For cell-based experiments except for LC-MS/MS, the perfect blinding could not be achieved because the allocating of cell groups, data collection, and data analysis were performed by the same researchers. For LC-MS/MS experiment, the investigator performed the experiment with blinded samples labeled 1-1, 1-2, ..., 4-3 (corresponding to triplicated samples for BTP-/hv-, BTP+/hv-, BTP-/hv+, and BTP+/hv+)

Reporting for specific materials, systems and methods

We require information from authors about some types of materials, experimental systems and methods used in many studies. Here, indicate whether each material, system or method listed is relevant to your study. If you are not sure if a list item applies to your research, read the appropriate section before selecting a response.

Materials & experimental systems

n/a	Involved in the study
☐	☒ Antibodies
☐	☒ Eukaryotic cell lines
☒	☐ Palaeontology and archaeology
☒	☐ Animals and other organisms
☒	☐ Clinical data
☒	☐ Dual use research of concern
☒	☐ Plants

Methods

n/a	Involved in the study
☒	☐ ChIP-seq
☐	☒ Flow cytometry
☒	☐ MRI-based neuroimaging

Antibodies

Antibodies used

Gasdermin D: NBP2-33422 (Novus Biologicals) WB - 1000:1
Caspase-1: ab207802 (Abcam) and PA5-29342 (Invitrogen) WB - 1000:1
Caspase-3: ab32351 (Abcam) WB - 1000:1
Caspase-4: A94799 (Antibodies) WB - 1000:1
Caspase-5: sc-393346 (Santa Cruz) WB - 500:1
IL-1beta: P420B (Invitrogen) WB - 1000:1
IL-18: PA5-79479 (Invitrogen) WB - 1000:1
Beta-actin: MA5-15739 (Invitrogen) WB - 1000:1
Anti-rabbit HRP: ab205718 (Abcam) WB - 3000:1
Anti-mouse HRP: 31430 (Invitrogen) WB - 5000:1

Validation

All antibody were commercially available. The manufacturer provides its validation on their website.
Gasdermin D: https://www.novusbio.com/products/gsdmdc1-antibody_nbp2-33422
Caspase-1: https://www.abcam.com/caspase-1-antibody-epr19672-ab207802.html
Caspase-1: https://www.thermofisher.com/antibody/product/Caspase-1-Antibody-Polyclonal/PA5-29342
Caspase-3: https://www.abcam.com/caspase-3-antibody-e87-ab32351.html
Caspase-4: https://www.antibodies.com/caspase-4-p20-cleaved-gln81-antibody-a94799
Caspase-5: https://www.scbt.com/p/caspase-5-p20-antibody-h-2
IL-1beta: https://www.thermofisher.com/antibody/product/IL-1-beta-Antibody-Polyclonal/P420B
IL-18: https://www.thermofisher.com/antibody/product/IL-18-Antibody-Polyclonal/PA5-79479
Beta-actin: https://www.thermofisher.com/antibody/product/beta-Actin-Loading-Control-Antibody-clone-BA3R-Monoclonal/MA5-15739
Anti-rabbit HRP: https://www.abcam.com/goat-rabbit-igg-hl-hrp-ab205718.html
Anti-mouse HRP: https://www.thermofisher.com/antibody/product/Goat-anti-Mouse-IgG-H-L-Secondary-Antibody-Polyclonal/31430

Eukaryotic cell lines

Policy information about cell lines and Sex and Gender in Research

Cell line source(s)

HeLa, Panc-1, A549, and Miapaca-2: ATCC
WT and GSDMD KO iBMDMs were immortalized from mouse (James Vince Lab, WEHI)

Authentication

commercial cell lines were authenticated by commercial vendors.
Example morphology, STP profiling, Karyotype, or other information about the cell lines are provided in website below
HeLa (ATCC, CCL-2): https://www.atcc.org/products/ccl-2
PANC-1 (ATCC, CRL-1469): https://www.atcc.org/products/crl-1469
A549 (ATCC, CCL-185): https://www.atcc.org/products/crm-ccl-185?nt=wobj-20-q
Miapaca-2 (ATCC, CRL-1420): https://www.atcc.org/products/crl-1420

GSDMD KO was authenticated by western blot (supplementary information Fig. 33)

Mycoplasma contamination

The cell lines were tested negative for mycoplasma contamination

Commonly misidentified lines
(See ICLAC register)

None.

Plants

Seed stocks	N/A
Novel plant genotypes	N/A
Authentication	N/A

Flow Cytometry

Plots

Confirm that:

- [x] The axis labels state the marker and fluorochrome used (e.g. CD4-FITC).
- [x] The axis scales are clearly visible. Include numbers along axes only for bottom left plot of group (a 'group' is an analysis of identical markers).
- [x] All plots are contour plots with outliers or pseudocolor plots.
- [x] A numerical value for number of cells or percentage (with statistics) is provided.

Methodology

Sample preparation	HeLa cells of 7–80% confluence in 6-well plate were treated with BTP (10 μM) for 2 h and further incubated with Rhod-2 AM (3 μM) and Ion K+ green-2 (40 μM) for an hour. Then, the cells were irradiated with blue LED (450 nm, 10 J·cm2). 2 hours after irradiation, the cells were detached using cell scrapper and the fluorescence of cell suspension was detected using flow cytometry (CytoFLEX S, Beckman, USA). Further information is provided in the supporting information.
Instrument	CytoFLEX S, Beckman, USA
Software	CytExpert software (ver. 2.4.0.28).
Cell population abundance	The abundance of the HeLa cell population within post-sort fractions is 10000. We selected experimental viable cells of reasonable size (positive FSC, intensity < 40 X 10^5) with internal complexity (positive SSC, intensity < 40 X 10^5) except for cell debris. The purity of the samples is above 80%
Gating strategy	The cells were selected by the preliminary FSC-A/SSC-A gates. Cells with positive FSC and positive SSC are selected, and discarded signals from cells with extremely high SSC (> 40 X 10^5) or FSC (> 40 X 10^5). For both of the mitochondrial Ca2+ assay (Rhod-2 assay) and intracellular K+ assay (ION K+ Green assay), we measured signals of dyes with Laser/filter combination of mCherry. We defined the cation-mobilized cells (BTP+/hv+) based on the histogram of control condition (BTP +/hv-).

- [x] Tick this box to confirm that a figure exemplifying the gating strategy is provided in the Supplementary Information.
